# Optimizing exercise prescriptions for older adults with mild cognitive impairment: a dose–response network meta-analysis of modality–dose combinations on executive function

**DOI:** 10.7717/peerj.21805

**Published:** 2026-10-20

**Authors:** Mingliang Song, Qiang Xiong, Xiaojin Qin

**Affiliations:** 1 School of Football, Wuhan Sports University, Wuhan, Hubei, China; 2 The College of Physical Education, Minnan Normal University, Zhangzhou, Fujian, China; 3 Nanfang College Guangzhou, Guangzhou, Guangdong, China

**Keywords:** Exercise, Prescription, Dose, Ageing, Mild cognitive impairment

## Abstract

**Objective:**

To use a Bayesian network dose–response meta-analysis to determine how overall exercise dose and different exercise modalities influence the three core domains of executive function-working memory, cognitive flexibility, and inhibitory control-in older adults with mild cognitive impairment.

**Methods:**

PubMed, Embase, Web of Science, and the Cochrane Library were searched through 10 November 2025 for randomized controlled trials. Two reviewers independently screened eligible studies. Studies were included if they enrolled adults aged ≥60 years with mild cognitive impairment, evaluated repeated exercise programmes, included usual care, low-intensity activity, or another exercise modality as a comparator, and reported at least one outcome of working memory, cognitive flexibility, or inhibitory control. Studies were excluded if participants had dementia or other neurological conditions affecting cognition, if only acute single-bout exercise was examined, if the study was non-randomized or non-peer-reviewed, if essential outcome data were unavailable, or if the Physiotherapy Evidence Database score was <4. Exercise dose was standardized as metabolic equivalents (METs)⋅min/week, and Bayesian model-based network meta-analysis was used to estimate non-linear overall and modality-specific dose–response relationships.

**Results:**

Thirty-eight studies met the eligibility criteria. Across all three executive-function dimensions, the overall dose–response curves exhibited a characteristic non-linear improvement pattern. Moderate exercise doses (approximately 300–1,000 METs⋅min/week) were associated with the most reliable gains, whereas higher-dose ranges showed plateaus or modest declines. Effective dose windows varied by modality and cognitive dimension: Exercise Joint Cognitive Training yielded the most consistent improvements in working memory, while Comprehensive Physical Training and Walking Aerobic Exercise demonstrated more stable effects on cognitive flexibility and inhibitory control. Sensitivity analyses supported the stability of the models. The certainty of evidence ranged from low to moderate, and publication bias was minimal.

**Conclusion:**

Exercise yields a non-linear dose–response relationship with executive function in older adults with mild cognitive impairment, with moderate doses producing the most robust improvements. Distinct optimal dose windows exist across cognitive dimensions and exercise modalities, offering a basis for precision exercise–cognition prescriptions. These findings support prioritizing dose-informed intervention strategies in ageing and cognitive-decline management and highlight the need for future trials adopting multi-dose gradient designs to validate optimal dose ranges.

## Introduction

Cognitive impairment has emerged as one of the fastest-growing public health challenges in the context of global population ageing  (Chen et al., 2023a). According to the World Health Organization, more than 55 million individuals currently live with some degree of cognitive decline, and this number is projected to increase substantially in the coming decades (Zhang et al., 2023; Langa & Levine, 2014). Mild cognitive impairment (MCI), positioned between normal ageing and dementia, is particularly prevalent among older adults, affecting nearly one-fifth of this population (Levy et al., 2022). MCI carries a notable risk of progression to dementia, with longitudinal evidence suggesting that more than half of individuals eventually transition to more severe cognitive deterioration  (Petersen et al., 2018). Accompanying deficits in memory and executive function can directly undermine daily functional independence and impose long-term burdens on families and healthcare systems (Choi et al., 2022; Raffin et al., 2023; Buele et al., 2024).

Despite representing an at-risk transitional state, MCI retains a degree of cognitive plasticity. Evidence indicates that executive function is among the most vulnerable domains yet remains responsive to appropriate interventions (Ornish et al., 2024; Mian et al., 2024). Executive function was selected as the primary cognitive domain because its core components-working memory, inhibitory control and cognitive flexibility-are essential for adaptive behaviour and everyday functioning (Corbo & Casagrande, 2022). Individuals with MCI commonly exhibit executive dysfunction, which has also been associated with poorer performance in instrumental activities of daily living and reduced functional independence (Lin et al., 2022). Therefore, clarifying exercise dose–response relationships across specific executive-function domains has direct clinical relevance for older adults with MCI. Pharmacological options for MCI remain limited. Acetylcholinesterase inhibitors have not been associated with slower cognitive decline compared with placebo (Hsu et al., 2025), whereas anti-amyloid monoclonal antibodies such as lecanemab and donanemab can slow clinical decline in patients with biomarker-confirmed early Alzheimer’s disease, including MCI due to Alzheimer’s disease  (Van Dyck et al., 2023). However, these treatments are associated with adverse events, particularly amyloid-related imaging abnormalities and infusion-related reactions (Van Dyck et al., 2023; Sims et al., 2023). Thus, safe and accessible non-pharmacological interventions such as exercise remain important. Consequently, exercise-based interventions-characterized by high safety and ease of implementation-have gained prominence as either substitutes for or complements to medication. A growing body of evidence demonstrates that exercise can produce meaningful improvements in executive function among individuals with MCI (Castellote-Caballero et al., 2024; Vints et al., 2024; Wang et al., 2024).

Over the past decade, trials examining the cognitive benefits of various exercise modalities have expanded substantially, including resistance training (Krootnark et al., 2024), aerobic exercise (Huang, Chen & Li, 2023), mind–body exercise (Chen et al., 2023b), multicomponent training (Li et al., 2022) and combined exercise–cognitive training (Buele et al., 2024). Although these interventions generally yield positive outcomes, heterogeneity in exercise frequency, intensity, session duration, total volume and intervention length-collectively reflecting the multidimensional construct of “exercise dose”-complicates cross-study comparisons. Moreover, most previous reviews have emphasised the binary question of “whether exercise is effective”, with limited attention to the dose–response patterns that ultimately determine clinical applicability and the development of actionable exercise prescriptions. This gap limits clinicians and researchers from determining dose thresholds supported by evidence, thereby hindering the translation of exercise interventions into MCI-specific prescription guidelines.

While dose-based exercise recommendations have been explored in other clinical populations such as stroke survivors (Sui & Wang, 2025), the MCI field still lacks an integrative synthesis capable of clarifying dose requirements and identifying differences across exercise modalities. This gap is critical because MCI represents a key window of opportunity during which cognitive decline may still be modifiable; yet most studies report only the presence or absence of improvements, without providing dose-level information that can inform clinical or community implementation. The absence of dose references confines exercise interventions to broad, non-specific recommendations, preventing their advancement toward prescriptive, scalable and evidence-informed practice, and ultimately restricting progress in precision non-pharmacological intervention strategies. Importantly, the present review focused specifically on the chronic adaptations associated with repeated, structured exercise programmes rather than the acute cognitive responses elicited by a single bout of exercise. Acute exercise effects are typically transient and reflect immediate physiological and neurocognitive responses, whereas repeated exercise exposure may induce cumulative and more sustained adaptations relevant to cognitive plasticity. Because the primary aim of this study was to identify dose ranges that could inform practical and longer-term exercise prescriptions for older adults with MCI, chronic intervention effects were considered more directly relevant to the clinical and community implementation of exercise programmes. To address this need, the present study applied a Bayesian dose–response network meta-analytic framework (Swinton, Schoenfeld & Murphy, 2024; Pan, Zheng & He, 2025) to synthesize evidence from randomized controlled trials and model the effects of distinct exercise modalities and modality–dose combinations on executive function in individuals with MCI. Using both direct and indirect comparisons, this approach yields more stable and coherent inference across heterogeneous exercise programmes. Executive function was further decomposed into its three core subdomains-working memory, cognitive flexibility and inhibitory control-to delineate domain-specific dose–response curves and identify optimal dose windows and superior modality combinations. This work aims to fill the critical evidence gap regarding dose-level exercise prescriptions for MCI, thereby providing a methodological and theoretical foundation for the development of precise, implementable and scalable non-pharmacological cognitive interventions.

## Method

### Study design and reporting standards

The overall study process followed the guidelines of the Preferred Reporting Items for Systematic Reviews and Meta-Analyses (PRISMA)  (Page et al., 2021). Prior to conducting the review, the study protocol was prospectively registered on the PROSPERO platform (CRD420251236616) to ensure methodological transparency and traceability. The research framework encompassed literature search, study screening, data extraction, risk-of-bias assessment and construction of the Bayesian dose–response network meta-analytic models.

### Literature search strategy

To systematically identify randomized controlled trials (RCTs) examining the effects of exercise interventions on executive function in older adults with mild cognitive impairment, comprehensive searches were performed across the Cochrane Library, Web of Science, Embase and PubMed. Search strategies combined controlled vocabulary terms (MeSH/Emtree) with free-text keywords, with database-specific adjustments applied to optimize sensitivity and specificity. All searches were independently conducted by Ming-liang Song and Qiang Xiong, who also manually screened the reference lists of relevant systematic and narrative reviews to minimize the risk of missing earlier or incompletely indexed studies.

### Eligibility criteria

Eligibility criteria were determined according to the PICOS framework:

(1) Population

Studies were required to include individuals aged 60 years or older with a diagnosis of mild cognitive impairment based on clinical evaluation or validated cognitive assessment tools. No restrictions were placed on sex, country or educational background. Individuals with neurological conditions known to affect cognition-including Parkinson’s disease, stroke and Alzheimer’s disease-were excluded to maintain consistency in the source of cognitive impairment.

(2) Intervention

Eligible interventions involved clearly defined, repeated exercise programmes designed to induce chronic training adaptations, with no restrictions on exercise modality. Studies examining only the acute effects of a single bout of exercise or cognitive outcomes measured exclusively as immediate post-exercise responses were not eligible. Interventions ranged from traditional exercise to virtual-reality-based programmes incorporating physical activity. To support the network structure required for network meta-analysis, differences in exercise modality between intervention and control groups were permitted (*e.g.*, resistance training *versus* aerobic exercise).

(3) Comparison

Control conditions included usual care or low-intensity activities such as stretching, relaxation, massage, health education or maintenance of daily routines. Controls could also include another exercise modality, enabling indirect comparisons across interventions.

(4) Outcomes

Studies were required to report at least one executive function outcome, including working memory, cognitive flexibility or inhibitory control, to allow incorporation into the dose–response models.

(5) Study design

Only randomized controlled trials were eligible; other study designs were excluded.

### Exclusion criteria

To ensure data quality and population consistency, studies were excluded if they met any of the following conditions: Ineligible population: Participants had progressed to dementia or had comorbid conditions that could independently cause cognitive impairment; or the control group did not consist of individuals with mild cognitive impairment. Unavailable data: Essential outcome data could not be extracted, and attempts to contact study authors for clarification were unsuccessful. Inappropriate publication type: Studies not peer-reviewed-including dissertations, conference abstracts, reviews, protocols and duplicate publications-were excluded. Insufficient methodological quality: Studies scoring below 4 on the revised Physiotherapy Evidence Database (PEDro) scale were excluded  (Chen, Du & Zhu, 2024).

### Data handling

During data processing, Ming-liang Song and Qiang Xiong independently screened all candidate studies according to the predefined inclusion and exclusion criteria and extracted the required information. Any disagreements were first resolved through discussion. If consensus could not be reached, Xiao-jin Qin acted as the independent referee and made the final decision.

Extracted data included the following categories: Study characteristics: first author, year of publication and country or region of study; Participant information: total sample size and demographic indicators such as mean age; Intervention details: exercise modality, training frequency, session duration, total intervention period and dose-related parameters expressed in metabolic equivalents (METs); Outcomes: pre–post executive function measures or raw data sufficient to compute effect sizes.

When means and standard deviations were not directly reported, estimates were derived from medians, interquartile ranges, ranges and sample sizes following established methods. Standard errors, confidence intervals or interquartile ranges were converted to standard deviations using standard formulae to ensure consistency across studies  (Cumpston et al., 2022). When multiple instruments were used to assess the same outcome domain, only the most representative measure was retained to avoid double-counting and undue weighting of individual studies  (Chen, Du & Zhu, 2024).

### Quality appraisal

Methodological quality for all included randomized controlled trials was assessed independently by two reviewers. Discrepancies were addressed through discussion, and unresolved disagreements were adjudicated by a third reviewer to maintain neutrality in the appraisal process. Methodological quality for all included randomized controlled trials was assessed independently by Ming-liang Song and Qiang Xiong. Discrepancies were first addressed through discussion, and any unresolved disagreements were adjudicated by Xiaojin Qin as the designated referee to maintain neutrality in the appraisal process.

Quality was evaluated using the PEDro scale, which comprises 11 items; the first item reflects external validity and is not included in the final score. The remaining 10 items contribute to a total score ranging from 0 to 10. Studies scoring below 4 were considered methodologically inadequate and excluded from the analysis. Scores of 4–5 indicated fair quality, 6–7 indicated moderate quality and scores of 8 or above were classified as high quality (Duan, Xiong & Chen, 2025). The PEDro scale has been widely applied in exercise science and rehabilitation research due to its strong discriminant validity and operational clarity, making it a suitable tool for evaluating the rigour and reporting quality of randomized controlled trials.

### Analytical framework and computational procedures

To characterize the dose–response patterns of exercise interventions on executive function in older adults with mild cognitive impairment, we employed a Bayesian network meta-regression framework (MBNMA) based on random-effects modelling (Mawdsley et al., 2016). Before formal modelling, the intervention network constructed from the included studies was examined to ensure a coherent and comparable structure. This step involved assessing whether the network formed closed loops and whether the assumptions of transitivity and consistency were reasonable, thereby avoiding inference under unmet theoretical conditions (Wheeler, Hickson & Waller, 2010; White et al., 2012).

Effect sizes were calculated as standardized mean differences (SMD; Hedges’ g) with corresponding 95% credible intervals (CrI), representing the comparative effects of different exercise modalities and dose levels on executive function (Etzioni & Kadane, 1995). Prior to model fitting, overall dose–outcome trends were visually inspected using dose–response scatterplots. Several candidate non-linear functional forms were then evaluated, including Emax models, restricted cubic spline models, quadratic polynomial functions and selected non-monotonic curve forms (Pedder et al., 2019). Model comparison considered the deviance information criterion (DIC), model complexity, residual patterns and overall heterogeneity. The quadratic polynomial model provided the most favourable balance between fit and parsimony and was therefore selected as the primary dose–response function for final inference (Evans, 2019).

For dose standardization, exercise modalities were first categorized based on intervention characteristics. MET values were assigned using the 2024 Compendium of Physical Activities (Herrmann et al., 2024) and METs compendium for older adults (Willis et al., 2024). Weekly training time was calculated as the product of session duration and weekly frequency, and converted into weekly energy expenditure (METs⋅min/week)  (Wasfy & Baggish, 2016; Ainsworth et al., 2011). Given the large variability in weekly energy expenditure across studies, directly modelling continuous dose values may compromise estimation stability. To enhance comparability and improve model convergence, weekly doses were grouped into a series of levels covering approximately 150–1,500 METs⋅min/week (Higgins et al., 2012).

For studies in which training frequency or session duration was not reported, missing parameters were replaced with the average values observed across the included trials. This approach ensured complete dose information while minimising systematic bias, as total weekly dose for each study was still derived from the available study-specific information, with only single missing components imputed using pooled means. All statistical analyses were conducted in R (version 4.4.2). The MBNMAdose package was used for model construction and posterior estimation, and ggplot2 was employed for visualising dose–response curves and their uncertainty intervals. Bayesian estimation was performed using the default vague prior distributions implemented in MBNMAdose, as no informative priors based on previous empirical evidence were prespecified. The study-specific baseline parameters and dose–response coefficients were assigned normal priors centred at zero with a precision of 0.0001, corresponding to *N*(0, 10,000) when expressed in terms of variance. For the random-effects component, the between-study standard deviation was assigned a vague uniform prior, SD ∼ Uniform(0, 520.081). These default vague priors were used to minimise the influence of prior assumptions and allow the posterior estimates to be predominantly informed by the observed data. Markov chain Monte Carlo estimation was conducted using three chains with 20,000 iterations per chain, with the first 10,000 iterations discarded as burn-in. Model convergence was assessed using the Gelman–Rubin potential scale reduction factor (Rhat), with values below 1.05 considered indicative of satisfactory convergence.

### Evidence grading and robustness verification

To ensure that the conclusions of this study were supported by credible and interpretable evidence, two reviewers independently assessed the strength of evidence for all included studies following a protocol established prior to data extraction. Any discrepancies were revisited through joint review of the source materials and scoring criteria. When disagreements persisted, a third reviewer-who had not participated in the initial evaluation-made the final judgement to preserve independence and impartiality in evidence grading. Evidence quality was appraised using the Grading of Recommendations Assessment, Development and Evaluation (GRADE) framework (Salanti et al., 2014). This system provides a structured and quantitative basis for evaluating the reliability and applicability of study findings, focusing on five domains: (1) risk of bias in study design and implementation; (2) consistency in the direction and magnitude of effects across studies; (3) directness of comparisons, including the match between interventions, populations and outcomes and the research question; (4) precision of effect estimates, considering confidence interval width and sample adequacy; and (5) potential publication or reporting bias. Based on these domains, each primary outcome was graded as “high”, “moderate”, “low” or “very low”, thereby indicating the reliability of evidence for clinical interpretation and future research.

Systematic application of the GRADE framework allowed us to delineate the robustness boundaries of the results and identify potential sources of uncertainty. This information supports the interpretation of sensitivity analyses, the formulation of dose-informed exercise recommendations and the design of future research aimed at refining optimal dose thresholds.

## Results

### Literature search

Using the predefined search strategy, a total of 20,271 records were retrieved from the Cochrane Library, Web of Science, PubMed and Embase as of 10 November 2025. After removal of duplicates and screening of titles and abstracts, most records were excluded due to mismatches in population, intervention type or study design. A smaller subset underwent full-text review. Following full-text assessment, 379 articles were excluded for reasons including inappropriate comparator conditions, non-MCI populations, duplicate publication, insufficient intervention duration, non-randomized designs or outcomes unrelated to executive function. In total, 38 studies met all eligibility criteria (Fig. 1). Manual searching yielded an additional 262 records; however, none met the inclusion criteria upon subsequent screening and full-text evaluation. Detailed characteristics of the 38 included studies are provided in Supplementary File 1.

**Figure 1:**
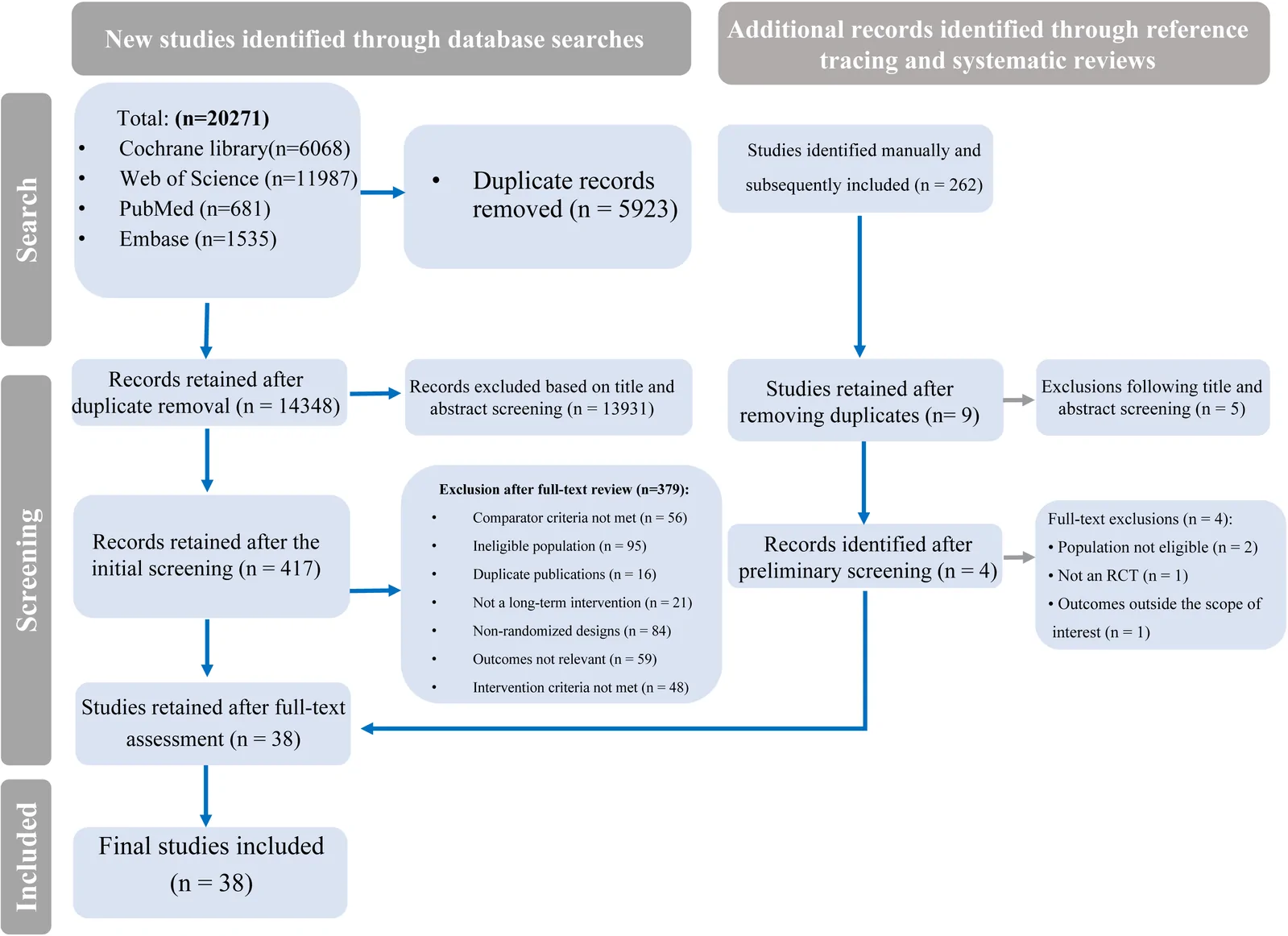
PRISMA 2020 flow diagram of study identification, screening, eligibility assessment, and inclusion. The flow diagram summarizes records identified through database searching and additional reference tracing, duplicate removal, title and abstract screening, full-text eligibility assessment, reasons for exclusion, and final study inclusion. RCT, randomized controlled trial; MCI, mild cognitive impairment.

### Characteristics of included studies

In total, 38 randomized controlled trials published between 2010 and 2025 were included (Table 1). All included studies enrolled older adults with MCI according to the diagnostic criteria or eligibility definitions specified in the original randomized controlled trials. Although the specific approaches to MCI ascertainment varied across studies, all participants met the MCI-related eligibility requirements predefined by the respective trials. Across studies, intervention periods were generally concentrated, with an average duration of approximately 10 weeks. The included trials covered a broad spectrum of exercise modalities commonly applied in cognitive interventions for older adults. Based on training characteristics and exercise attributes, interventions were categorized into seven types: resistance training, virtual reality training, aerobic exercise, mind-body exercise, comprehensive physical training, functional training and exercise joint cognitive training. These programmes were typically designed and implemented by research teams with expertise in exercise science, rehabilitation or related fields, ensuring structured and operationalizable interventions that facilitated comparison of dose–response relationships across studies. Overall, the included studies demonstrated strong consistency in participant characteristics, diagnostic criteria and intervention classification, providing a robust evidence base for the subsequent Bayesian network meta-analysis and dose–response modelling.

### Methodological quality

The methodological quality of all 38 included trials was assessed using the PEDro scale (Table 2). Scores ranged from 5 to 8, with most studies scoring between 6 and 7, indicating overall moderate-to-high quality. Nearly all trials reported clear eligibility criteria, random allocation and baseline comparability. As is typical in exercise-intervention research, participant and therapist blinding was rarely feasible, resulting in consistently low scores on these items. Assessor blinding (item A7) was implemented in most studies. Reporting of primary outcome measures, statistical comparisons and variability indices was generally adequate, although intention-to-treat analysis (A9) remained infrequent. Overall, the methodological quality across studies was sufficiently consistent to support the subsequent network meta-analysis and dose–response modelling.

**Table 1:** Characteristics of the included studies.

First author (year, country)	Type	E (*n*)/C (*n*)	Age (E/C)	E	C	Cycle, weekly frequency, time (min)		Outcome indicator
						E	C	
Juliana Yumi Tizon Kasai (2010, Brazil)	S-T	13/13	73.54/74.54	MBE	CTH	24/2/60	24/2/60	DST
Linda C.W. Lam (2010, China)	S-T	171/218	77.2 ± 6.3/78.3 ± 6.6	MBE	CTH	10/3/30	10/3/30	DST, TMT
Takao Suzuki (2012, Japan)	T	25/25	75.3 ± 7.5/76.8 ± 6.8	CPT	CTH	24/2/60	NA	Letter verbal fluency test, ST, DST
Jennifer C. Davis (2013, UK)	T	28/30/28	75.0 ± 3.7/75.5 ± 3.5/74.1 ± 3.6	RT	Walking AE/CPT	24/2/60	24/2/60	ST
Deborah E. Barnes (2013, United States)	S-Fo	32/31	73.9 ± 6.3/71.1 ± 5.5	CPT	CTH	12/3/60	12/3/60	TMT, DST, Flanker test
Thomas M. Dannhauser (2014, UK)	T	43	73.9 ± 8.3	Walking AE	CT	12/3/45	12/3/45	TMT
Linda Chiu-wa Lam (2015, China)	S-Fo	145/132/147/131	74.4 ± 6.4/76.3 ± 6.6/75.5 ± 6.7/75.4 ± 6.1	EJCT	CTH/MBE/CTH	16/NA/NA	16/NA/NA	Category verbal fluency test
Jiaojiao Lü (2016, China)	S-T	22/23	69.00 ± 3.83/70.43 ± 5.53	RT	CTH	12/3/60	12/3/60	ST, TMT
Jungha Hwang (2017, Korea)	T	12/12	74.1 ± 6.0/70.1 ± 5.3	VRT	CTH	NA/total 20/30	NA/total 20/30	Visual span test, word color test
Harris A. Eyre (2017, United States)	Triple-T	38/41	68.1 ± 8.7/67.6 ± 8.0	MBE	CTH	48/NA/60	NA	Rey-Osterrieth complex figure test, TMT, ST
Takehiko Doi (2017, Japan)	S-Th	67/67	76.0 ± 4.9/75.7 ± 4.1	EJCT	CTH	40/NA/60	40/NA/60	Story and word memory tests, TMT
Somporn Sungkarat (2018, Thailand)	S-T	33/33	68.3 ± 6.7/67.5 ± 7.3	MBE	CTH	12/3/30	NA	DST, TMT
Soon-Gook Hong (2018, Korea)	T	12/10	78.33 ± 63.21/78.33 ± 65.50	RT	CTH	12/2/40	12/2/40	Controlled oral word test, ST, DST
DH YOON (2018, Korea)	T	20/23	73.82 ± 4.37/74.03 ± 4.27	RT	CTH	16/3/40	16/3/40	TMT, DST
Arsalan Damirchi (2018, Iran)	Th	13/11/11	68.81 ± 3.68/67.90 ± 3.75/67.76 ± 4.69	EJCT	CPT/CTH	8/NA/60	8/NA/60	DST, ST
Donnezan, Laure Combourieu (2018, France)	Fo	18/16/21/14	77.1 ± 1.4/76.3 ± 1.5/75.2 ± 1.3/79.2 ± 4	EJCT	AE/CTH/CTH	12/2/60	12/2/60	DST, ST
Eunhee Park (2019, Korea)	S-T	10/11	70.60 ± 4.29/73.36 ± 5.50	VRT	CT	6/3/30	6/3/30	TMT
Ying-Yi Liao (2019, Taiwan)	S-T	18/16	75.5 ± 5.2/73.1 ± 6.8	VRT	EJCT	12/3/60	12/3/60	TMT, ST
Lawla L.F. Law (2019, China)	S-Fo	16/14/15/14	77.94 ± 6.11/71.57 ± 7.43/76.93 ± 6.79/75.14 ± 8.53	FT	CPT/CTH/CTH	NA/8/40	NA/8/40	TMT
Rui Xia (2019, China)	T	23/23/23	65.79 ± 4.35/64.88 ± 3.30/65.86 ± 5.28	MBE	Walking AE/CTH	24/3/60	24/3/60	ST, Test of attention performance, DST
Ji-Su Park (2020, Korea)	T	18/17	75.8 ± 8.5/77.2 ± 7.2	VRT	CTH	6/5/60	6/5/60	TMT, DST
Rosa Manenti (2020, United States)	S-Th	18/17	65 years and above	VRT	CTH	12/3/NA	12/3/NA	TMT
Ngeemasara Thapa (2020, Korea)	T	34/34	72.6 ± 5.4/72.7 ± 5.6	VRT	CTH	8/3/100	8/1/50	TMT
Polly W.C. Li (2022, China)	S-T	116/113	73.93 ± 7.40/74.83 ± 7.57	CPT	CTH	8/NA/NA	NA	TMT, DST
Rui Xia (2023, China)	S-T	45/45/45	66.16 ± 4.16/66.08 ± 4.28/65.41 ± 4.90	MBE	AE/CTH	24/3/60	24/3/60	ST, DST, TMT
Fairchild (2023, United States)	S-T	36/36	71.0 ± 8.8/73.7 ± 10.1)	CPT	CTH	24/3/50	24/3/50	ST, DST, TMT
Heng Wang (2024, China)	T	30/30	60–69	MBE	CTH	12/4/40	NA	ST
Kitsana Krootnark (2024, Thailand)	S-T	30/30/30	68.60 ± 4.86/68.70 ± 4.72/69.70 ± 5.55	Walking AE/RT	CTH	12/5/30	12/5/30	TMT, DST
Saeed (2024, Pakistan)	S-Fo	21/21/23/22	72.52 ± 7.38/64.83 ± 4.88/69.72 ± 8.07/72.32 ± 7.74	VRT	CTH	8/3/40	8/3/40	ST, TMT, DST
Meixiang Fan (2024, China)	S-T	59/57	66.3 ± 4.4/67.8 ± 5.1	EJCT	CTH	12/5/40	12/5/30	DST, TMT
Baker (2025, United States)	S-T	148/148	74.3 ± 5.7/74.7 ± 6.2	Walking AE	CTH	72/4/45	72/4/45	TMT, DST
Wing Keung (2025, China)	T	9/9	73.67 ± 6.144/80.00 ± 4.359	VRT	MBE	8/2/45	8/2/45	TMT
Sutaya Meekum (2025, Thailand)	S-T	18/18	68.39 ± 3.35/69.68 ± 3.85	Water sport	EJCT	12/3/60	12/3/60	ST, TMT, DST
Muñoz-Perete (2025, Spain)	T	40/41	70.90 ± 4.24/71.44 ± 5.21	EJCT	CTH	12/2/45	12/2/45	DST, TMT
Salviano Resende-Silva (2025, Brazil)	S-T	28/22/18	67.5 ± 4.8/66.4 ± 4.6/67.5 ± 4.6	FT/CPT	CTH	16/3/50	16/3/50	DST
Marcelina Sánchez-Alcalá (2025, Spain)	S-T	47/45	71.43 ± 2.97/72.24 ± 2.92	MBE	CTH	12/2/60	12/2/60	TMT
Suhaas Penukonda (2025, United States)	S-T	16/11	66.2 ± 7.1/66.4 ± 6.9	High intensity walking AE	Low intensity walking AE	52/3/30	52/3/30	TMT, Word interference test
Hong Yu (2025, China)	S-T	36/36	80.75 ± 5.27/81.86 ± 5.31	EJCT	CTH	24/4/25	24/4/25	DST, TMT

**Notes.**

S Single blind T Two arm Th Three arm Fo Four arm NA not available E experimental group C control group RT Resistance training VRT Virtual reality training AE Aerobic exercise MBE Mind-body exercise CT Cognitive training CPT Comprehensive physical training FT Functional Training EJCT Exercise Joint Cognitive Training CTH Conventional therapy ST Stroop task TMT Trail-Making Task DST Digit Span Test

**Table 2:** Methodological quality assessment of the included studies based on the PEDro scale.

Study	A1	A2	A3	A4	A5	A6	A7	A8	A9	A10	A11	Total
Juliana Yumi Tizon Kasai (2010, Brazil)	1	1	0	1	0	0	1	1	0	1	1	6
Linda C.W. Lam (2010, China)	1	1	1	1	0	0	1	1	1	1	1	8
Takao Suzuki (2012, Japan)	1	1	0	1	0	0	0	1	1	1	1	6
Jennifer C. Davis (2013, UK)	1	1	0	1	0	0	0	1	1	1	1	6
Deborah E. Barnes (2013, United States)	1	1	1	1	1	0	0	1	1	1	1	8
Thomas M. Dannhauser (2014, UK)	1	1	0	1	0	0	0	1	0	1	1	5
Linda Chiu-wa Lam (2015, China)	1	1	0	1	0	0	1	1	1	1	1	7
Jiaojiao Lü (2016, China)	1	1	1	1	0	0	1	1	0	1	1	7
Jungha Hwang (2017, Korea)	1	1	0	1	0	0	0	1	1	1	1	6
Harris A. Eyre (2017, United States)	1	1	0	1	1	1	1	1	0	1	1	8
Takehiko Doi (2017, Japan)	1	1	0	1	1	0	0	1	1	1	1	7
Somporn Sungkarat (2018, Thailand)	1	1	1	1	0	0	1	1	1	1	1	8
Soon-Gook Hong (2018, Korea)	1	1	0	1	0	0	0	1	0	1	1	5
D.H. YOON (2018, Korea)	1	1	0	1	0	0	0	1	0	1	1	5
Arsalan Damirchi (2018, Iran)	1	1	0	1	0	0	0	1	0	1	1	5
Donnezan, Laure Combourieu (2018, France)	1	1	0	1	0	0	0	1	0	1	1	5
Eunhee Park (2019, Korea)	1	1	0	1	0	0	1	1	0	1	1	6
Ying-Yi Liao (2019, Taiwan)	1	1	1	1	0	0	1	0	0	1	1	6
Lawla L.F. Law (2019, China)	1	1	1	1	0	0	0	1	0	1	1	6
Rui Xia (2019, China)	1	1	0	1	0	0	1	1	1	1	1	7
Ji-Su Park (2020, Korea)	1	1	1	1	0	0	1	0	0	1	1	6
Rosa Manenti (2020, United States)	1	1	1	1	0	0	0	1	0	1	1	6
Ngeemasara Thapa (2020, Korea)	1	1	1	1	0	0	1	1	1	1	1	8
Polly W.C. Li (2022, China)	1	1	0	1	0	0	0	1	1	1	1	6
Rui Xia (2023, China)	1	1	0	1	0	0	1	1	0	1	1	6
Fairchild (2023, United States)	1	1	0	1	0	0	1	1	0	1	1	6
Heng Wang (2024, China)	1	1	0	1	0	0	0	1	0	1	1	5
Kitsana Krootnark (2024, Thailand)	1	1	1	1	0	0	1	1	0	1	1	7
Saeed (2024, Pakistan)	1	1	0	1	0	0	1	1	0	1	1	6
Meixiang Fan (2024, China)	1	1	0	1	0	0	1	1	1	1	1	7
Baker (2025, United States)	1	1	1	1	0	0	1	1	1	1	1	8
Wing Keung (2025, China)	1	1	0	1	0	0	0	1	0	1	1	5
Sutaya Meekum (2025, Thailand)	1	1	0	1	0	0	1	1	0	1	1	6
Muñoz-Perete (2025, Spain)	1	1	1	1	0	0	0	1	0	1	1	6
Salviano Resende-Silva (2025, Brazil)	1	1	1	1	0	0	1	1	0	1	1	7
Marcelina Sánchez-Alcalá (2025, Spain)	1	1	0	1	0	0	1	1	0	1	1	6
Suhaas Penukonda (2025, United States)	1	1	0	1	0	0	1	1	0	1	1	6
Hong Yu (2025, China)	1	1	0	1	0	0	1	1	0	1	1	6

**Notes.**

PEDro items were scored dichotomously: 1, the criterion was clearly satisfied; 0, the criterion was not satisfied or was insufficiently reported. Item A1 (eligibility criteria) evaluates external validity and is not included in the total PEDro score. Items A2–A11 each contribute one point to the total score, yielding a maximum score of 10. A1, eligibility criteria specified; A2, random allocation; A3, concealed allocation; A4, baseline comparability; A5, participant blinding; A6, therapist blinding; A7, assessor blinding; A8, adequate follow-up (>85%); A9, intention-to-treat analysis; A10, between-group statistical comparisons; A11, point estimates and measures of variability.

### Consistency of network models

Consistency analyses demonstrated that the network models were stable across all comparisons. Posterior distributions of direct and indirect estimates showed substantial overlap across multiple sources of evidence (Supplemental Tables S1–S3 and S7–S9), with no indication of systematic divergence or conflicting signals. These findings suggest that comparisons among exercise modalities and dose levels were coherent within the shared analytical framework. No meaningful inconsistency was detected across the full network, indicating that the model structure exhibited strong global consistency. All principal Bayesian models demonstrated satisfactory convergence, with Rhat values below 1.01 for all monitored parameters. The maximum Rhat was 1.001457 for the overall exercise-dose models and 1.002550 for the exercise modality–dose models, providing further evidence of satisfactory MCMC convergence.

### Meta-analysis results

#### Dose–response effects of overall exercise dose on executive function

For cognitive flexibility, overall exercise dose produced small-to-moderate improvements across the dose range (Fig. 2; Figs. S1–S3; Tables S4–S6). At approximately 670 METs⋅min/week, the estimated effect size was 0.25 (95% CrI [0.073–0.524]). The dose–response curve showed a progressive increase in effect with rising dose, peaking at around 1,000 METs⋅min/week (mean ≈ 0.26; 95% CrI [0.051–0.534]). Credible intervals remained above zero throughout this moderate-dose range (≈300–1,000 METs⋅min/week). Beyond this range, the effect did not increase further; doses exceeding 1,200 METs⋅min/week showed attenuation and widening credible intervals, with lower bounds crossing zero, indicating greater uncertainty at higher doses.

**Figure 2:**
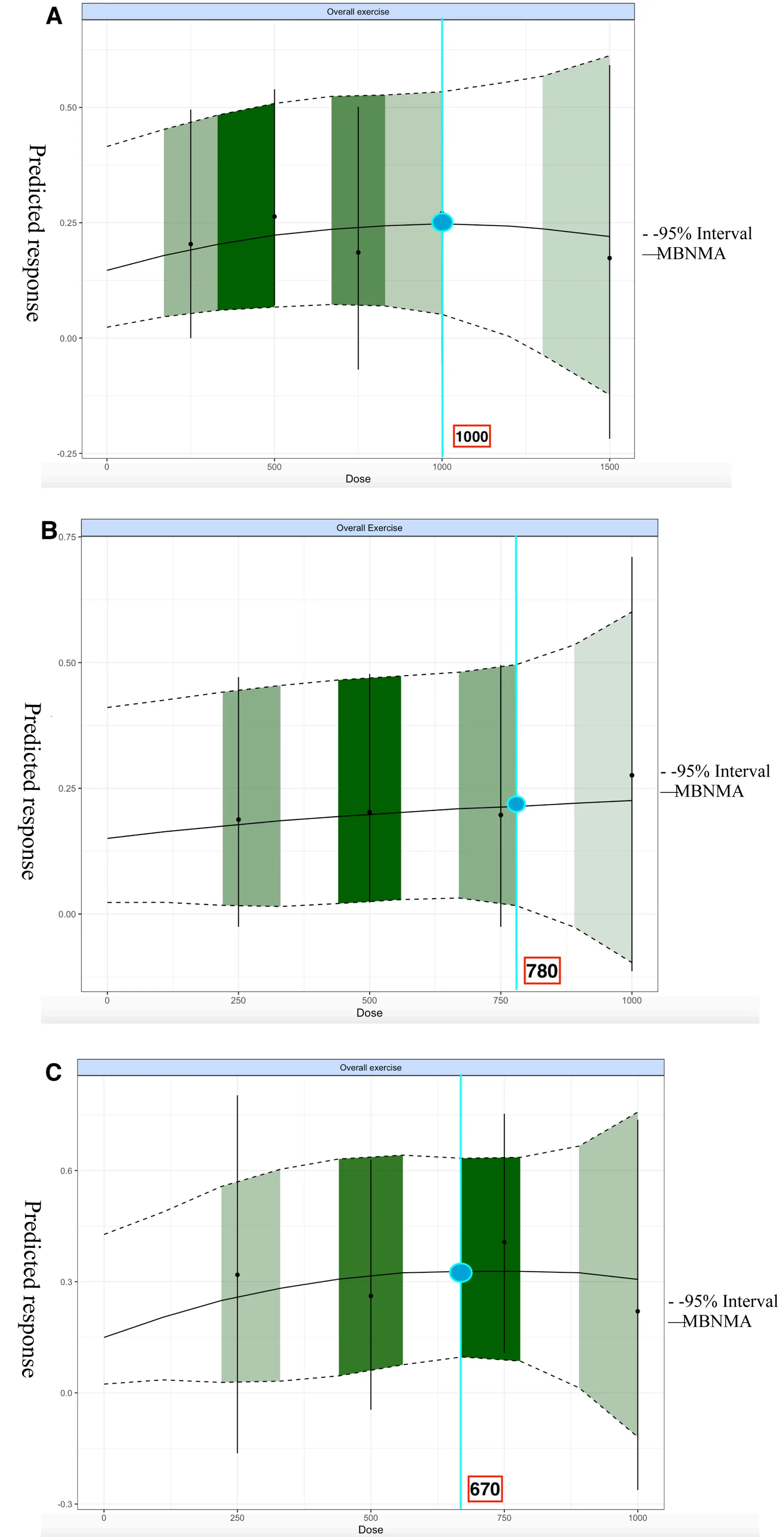
Bayesian dose–response relationships between overall weekly exercise dose and executive-function subdomains in older adults with mild cognitive impairment. (A) Cognitive flexibility; (B) working memory; and (C) inhibitory control. Exercise dose represents cumulative weekly exercise exposure expressed as metabolic equivalent task minutes per week (METs⋅min/week), calculated by multiplying the assigned MET value by session duration (min) and weekly exercise frequency. Predicted response represents the posterior mean standardized mean difference (SMD; Hedges’ g) relative to the comparator condition, with positive values indicating greater improvement in executive function. The solid curve represents the posterior mean dose–response relationship estimated by model-based network meta-analysis (MBNMA), and the dashed curves represent the corresponding 95% credible interval (CrI). Green shading indicates dose regions represented by observed data in the included studies; darker green indicates a greater number of studies contributing observed dose information at that location and does not represent a larger treatment effect or greater statistical significance. MET, metabolic equivalent task; SMD, standardized mean difference; CrI, credible interval; MBNMA, model-based network meta-analysis.

For working memory, overall dose yielded small but stable improvements. At 560 METs⋅min/week, the estimated effect size was 0.214 (95% CrI [0.029–0.473]). The effect increased gradually with dose and reached its highest value at approximately 780 METs⋅min/week (mean = 0.225; 95% CrI [0.017–0.496]). The most stable and statistically credible improvements were observed within the moderate-dose range (≈200–780 METs⋅min/week). Above approximately 780–890 METs⋅min/week, the effect became less consistent, and lower credible bounds crossed zero, indicating reduced certainty in the high-dose range.

For inhibitory control, overall dose produced moderate and statistically robust improvements. At 560 METs⋅min/week, the effect size was 0.331 (95% CrI [0.076–0.641]). The dose–response curve showed a steady increase in effect with higher dose, with a peak at around 670 METs⋅min/week (mean = 0.338; 95% CrI [0.097–0.633]). Stable and statistically reliable improvements were observed within the moderate-dose window (≈330–780 METs⋅min/week). Beyond this range (≈890–1,000 METs⋅min/week), effect growth was not maintained and credible intervals crossed zero, suggesting increased uncertainty at higher doses.

#### Dose–response effects of exercise modality–dose combinations on executive function

Across modality-specific dose–response models (Fig. 3; Tables S10–S12; Figs. S4–S6), only a limited number of modality–dose combinations demonstrated statistically significant effects on cognitive flexibility, defined as 95% CrIs not crossing zero. For comprehensive physical training, only doses of 670 and 780 MET⋅min/week showed entirely positive credible intervals, with the largest improvement observed at 670 MET⋅min/week. For walking aerobic exercise, significant effects were concentrated in the 420–500 MET⋅min/week range, with both doses exhibiting stable and relatively larger improvements. Other modalities, including exercise joint cognitive training (EJCT), mind–body exercise and resistance training, exhibited 95% CrIs that crossed zero across all doses, indicating the absence of consistent significant effects for cognitive flexibility.

**Figure 3:**
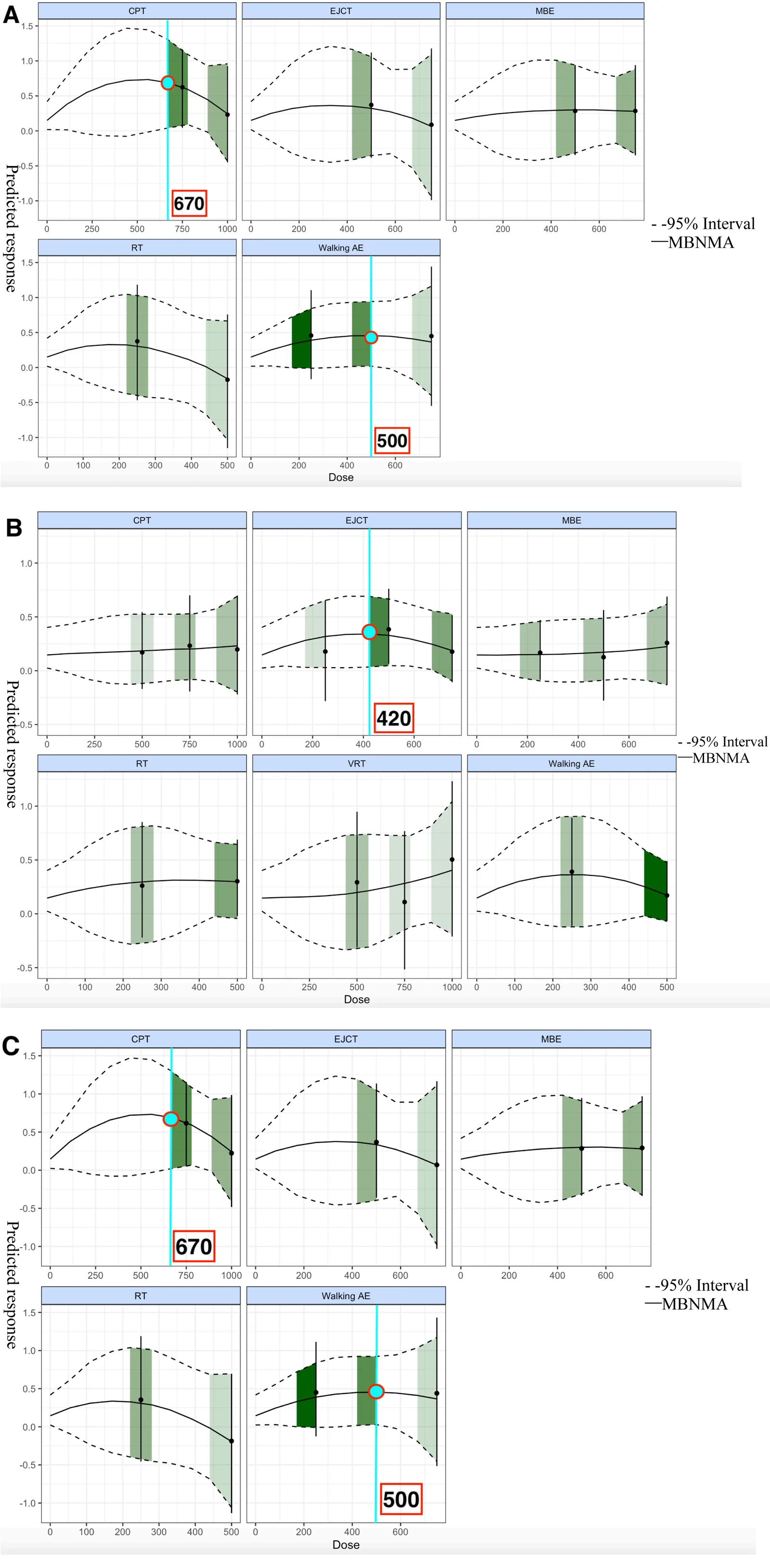
Dose–response relationships between exercise modality–dose combinations and executive function in older adults with mild cognitive impairment. A represents cognitive flexibility, B working memory, and C inhibitory control. The *x*-axis represents exercise dose (METs⋅min/week), and the *y*-axis represents the predicted response. The solid black line indicates the dose–response curve estimated using model-based network meta-analysis (MBNMA), and the dashed black lines represent the 95% credible interval (CrI). Black points indicate effect estimates at specific dose levels, with vertical black lines showing the corresponding uncertainty intervals. Green shaded areas indicate the distribution and density of available evidence across dose ranges. The cyan vertical line marks the estimated optimal exercise dose, the red open circle indicates the predicted effect at that dose, and the boxed number denotes the optimal dose value. Abbreviations: CPT, Comprehensive Physical Training; EJCT, Exercise Joint Cognitive Training; MBE, Mind–Body Exercise; RT, Resistance Training; VRT, Virtual Reality Training; Walking AE, Walking Aerobic Exercise; METs, metabolic equivalents; MBNMA, model-based network meta-analysis; CrI, credible interval.

For working memory, EJCT was the only modality showing statistically significant improvements. Its effective dose range spanned 83–580 MET⋅min/week, reaching the highest effect at approximately 420 MET⋅min/week (mean ≈ 0.34, with the lower bound of the 95% CrI remaining above zero). These results indicate that, among all modalities assessed, only EJCT demonstrated a clear and stable dose–response relationship for working memory.

For inhibitory control, significant dose–response effects were observed only for comprehensive physical training and walking aerobic exercise. Comprehensive physical training showed a stable non-linear improvement across 110–780 MET⋅min/week, with 670 MET⋅min/week producing the strongest effect and all credible intervals remaining above zero. Walking aerobic exercise also demonstrated consistent positive effects across 83–580 MET⋅min/week, reaching a peak at approximately 500 MET⋅min/week.

### Certainty of evidence

According to the GRADE assessments (Table S13), the certainty of evidence across executive-function outcomes ranged from low to moderate. Overall executive function, supported by 38 studies, was downgraded for risk of bias and imprecision due to limited blinding and relatively small sample sizes, but retained a moderate level of certainty. Working memory, informed by 25 studies, was also rated as moderate, reflecting methodological heterogeneity and wide credible intervals. Cognitive flexibility, assessed in 30 studies, showed greater variability in effect estimates, resulting in downgrades for inconsistency and imprecision and yielding low-to-moderate certainty. Inhibitory control was supported by fewer trials (17), with variation in effect direction leading to a low certainty rating. Although potential publication bias was detected in several domains, these signals did not alter the overall certainty judgments. Collectively, the current evidence base remains adequate to support dose–response inference, although further high-quality research is needed to strengthen certainty.

### Robustness analyses

Robustness of the dose–response relationships was examined across three domains: model structure, study quality and data perturbation. First, comparisons among multiple functional forms indicated that the quadratic model provided the most favourable balance of fit and interpretability and was therefore retained as the primary analytical framework. Second, sensitivity analyses excluding studies at high risk of bias produced dose–response curves with trends and key dose ranges that were highly consistent with the main analyses, suggesting that lower-quality studies did not materially influence the findings. Third, leave-one-out analyses were conducted to assess the influence of individual studies. Removal of any single study did not meaningfully alter effect directions or the dose levels associated with peak effects. Taken together, these results demonstrate that the dose–response models remained stable under a range of methodological perturbations, supporting the reliability and generalizability of the findings.

### Publication bias across executive-function domains

In the domain of working memory, Egger’s test indicated significant funnel-plot asymmetry (*z* = 2.425, *p* = 0.015). The trim-and-fill method suggested one potentially missing study; however, the adjusted effect size remained significant (0.206, 95% CI [0.122–0.290]), indicating limited influence of potential bias. For cognitive flexibility, Egger’s test also indicated significant asymmetry (*z* = 3.684, *p* < .001), and trim-and-fill procedures estimated nine missing studies. Despite substantial heterogeneity (*I*^2^ = 72.04%), the adjusted effect size remained robust (0.167, 95% CI [0.104–0.230]). By contrast, no evidence of publication bias was detected for inhibitory control (*z* = 0.839, *p* = 0.401), and the estimated intercept was non-significant, suggesting symmetric and stable effect estimates. Overall, although some domains exhibited signatures of small-study effects, trim-and-fill adjustments produced minimal changes in effect sizes, and conclusions remained stable across all executive-function outcomes (Fig. 4).

**Figure 4:**
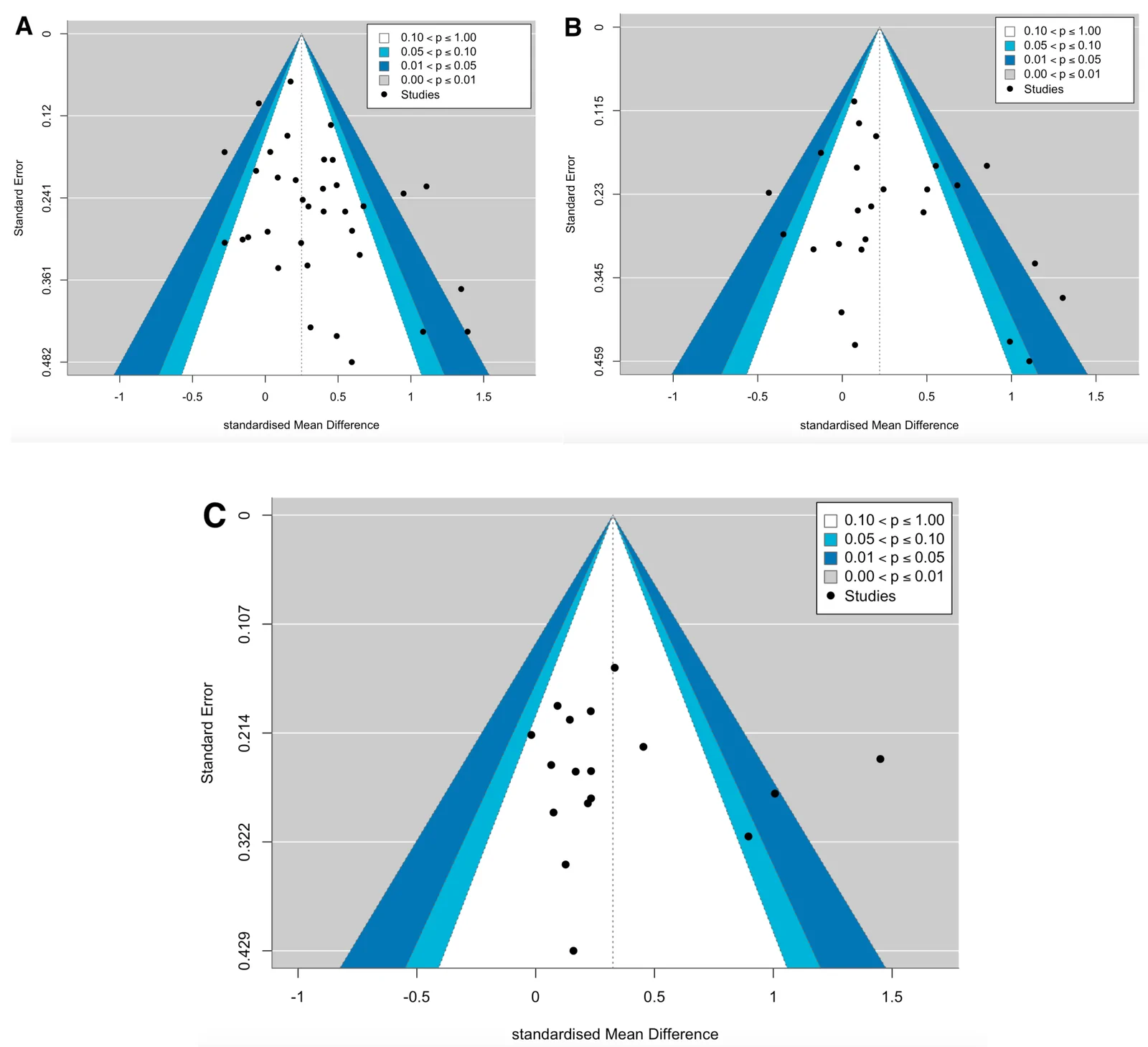
Contour-enhanced funnel plots for the assessment of publication bias across executive-function subdomains. (A) Working memory; (B) cognitive flexibility; and (C) inhibitory control. Each black point represents an individual study. The *x*-axis represents the standardized mean difference (SMD; Hedges’ g), and the *y*-axis represents its standard error (SE). The vertical reference line indicates the pooled effect estimate. Shaded contours denote regions of two-sided statistical significance around the null effect: *p* ≥ 0.10, 0.05 ≤ *p* < 0.10, 0.01 ≤ *p* < 0.05, and *p* < 0.01, as indicated in the legend. The contour-enhanced presentation assists in distinguishing funnel-plot asymmetry potentially related to publication bias from asymmetry arising from other sources. SMD, standardized mean difference; SE, standard error.

## Discussion

### Key findings

Using a Bayesian dose–response modelling framework, this study systematically examined the non-linear associations between overall exercise dose, modality–dose combinations and the three core dimensions of executive function. The findings revealed clear and dimension-specific response patterns. Across the overall dose analysis, cognitive flexibility, working memory and inhibitory control all demonstrated the most stable and statistically credible improvements within the moderate-dose range of approximately 300–1,000 METs⋅min/week. However, the peak dose and magnitude of improvement varied across dimensions: cognitive flexibility reached its maximum effect at around 1,000 METs⋅min/week, working memory peaked at approximately 780 METs⋅min/week and inhibitory control showed the strongest improvement at around 670 METs⋅min/week. Notably, when total exercise dose exceeded roughly 780–1,200 METs⋅min/week, improvements plateaued, credible intervals widened and lower bounds frequently crossed zero, indicating increased uncertainty in higher-dose ranges.

Modality-specific analyses further demonstrated that only a subset of exercise modalities produced significant effects within specific dose windows. Comprehensive physical training and walking aerobic exercise exhibited stable and statistically credible dose ranges for both cognitive flexibility and inhibitory control. In contrast, working memory displayed a distinct pattern: only exercise joint cognitive training (EJCT) showed a clear dose–response relationship, with an optimal dose around 420 METs⋅min/week. Collectively, these results underscore the differential dose sensitivity across executive-function dimensions and highlight that exercise modalities do not exert uniform effects across all dose levels. Instead, the observed efficacy is both dose-specific and modality-dependent, emphasising the importance of constructing precision exercise prescriptions aligned with the cognitive domain targeted.

### Contextualising the findings within prior evidence

The present study extends existing evidence by refining the relationship between exercise dose and executive-function improvement. Prior systematic reviews consistently concluded that exercise benefits executive function; however, most defined “exercise dose” using single components such as intensity or frequency, without quantifying dose thresholds or distinguishing dose sensitivity across cognitive dimensions. By employing non-linear Bayesian modelling, this study corroborates the commonly observed pattern that “moderate exercise dose yields the most robust effects”, while additionally quantifying the optimal dose ranges for cognitive flexibility, working memory and inhibitory control. The resulting dose-differentiated profiles reveal domain-specific peaks that were not captured in earlier analyses. This non-linear pattern is broadly consistent with Gallardo-Gómez et al. (2022), who reported that approximately 724 METs⋅min/week was associated with clinically relevant cognitive improvement in older adults, whereas benefits became less clear beyond 1,200 METs⋅min/week. Although their analysis focused on global cognition in adults aged ≥50 years rather than executive function specifically in individuals with MCI, the similar pattern supports the presence of an optimal intermediate exercise-dose range rather than a simple linear “more-is-better” relationship.

Moreover, previous research often attributed variability in cognitive outcomes to differences in exercise modality, yet few studies disentangled whether modality-specific benefits were attributable to the exercise itself or to the dose at which it was administered. Our findings indicate that only EJCT, comprehensive physical training and walking aerobic exercise demonstrate stable and credible effects within defined dose ranges, whereas other modalities-such as mind–body exercise or resistance training-did not produce consistent peaks or statistically significant intervals across doses. This pattern provides a theoretical explanation for the heterogeneous results reported in earlier meta-analyses, suggesting that modality effects are contingent on underlying differences in cognitive demands, motor complexity and energetic load. Consequently, the findings highlight the necessity of pairing modality with appropriate dose parameters when designing precision exercise–cognition interventions.

### Mechanistic insights into the dose–response relationship

The less consistent effects observed at lower doses may reflect an insufficient cumulative exercise stimulus to produce measurable cognitive adaptations. This interpretation is broadly consistent with Gallardo-Gómez et al. (2022), who also reported a non-linear dose–response relationship and identified an intermediate exercise dose as being associated with clinically relevant cognitive benefits. The non-linear pattern observed in this study-characterised by the most stable improvements at moderate doses and diminished gains at higher doses-is consistent with neurophysiological mechanisms proposed in recent reviews on physical activity and brain health. Moderate-intensity exercise has been shown to optimally enhance cerebral blood flow and oxygenation in the prefrontal cortex and hippocampus, as well as to increase the expression of brain-derived neurotrophic factor (BDNF), thereby supporting executive control and adaptive strategy updating (Boa Sorte Silva et al., 2024; Huang et al., 2022; Griebler et al., 2022; Brush et al., 2016). As exercise load increases further, metabolic demands and neural resource consumption also intensify, potentially creating competition among attentional resources, working-memory capacity and inhibitory processes, which may attenuate the benefits of higher exercise doses (Mcmorris, 2021; Brush et al., 2016; Weng et al., 2015). The non-linear dose–response pattern may reflect an “optimal physiological activation zone” of the prefrontal cortex. Moderate exercise appears to promote favourable conditions-enhanced cerebral perfusion, improved oxygen availability and increased neurotransmitter release-that allow executive-function networks to operate with maximal efficiency. When cumulative dose increases beyond this optimal window, rising metabolic load and fatigue may reduce available neural resources, resulting in plateauing or diminishing effects (Chang et al., 2012; Furley, Schütz & Wood, 2025).

Differences in the peak dose across executive-function dimensions may also reflect distinct neural resource requirements. Inhibitory control, which relies on circuits that are readily engaged by moderate activation, reached its peak at relatively lower doses. By contrast, working memory and cognitive flexibility involve higher energetic demands and greater requirements for large-scale network integration, potentially explaining their higher optimal doses (Chang et al., 2012; Furley, Schütz & Wood, 2025; Cantelon & Giles, 2021). Modality-specific findings further align with established neural mechanisms. EJCT demonstrated the most clearly defined effective dose range for working memory, consistent with its dual-load structure that simultaneously engages motor execution and higher-order cognitive processing (Chang et al., 2012; Cantelon & Giles, 2021; Tsai et al., 2024; Petrigna et al., 2025). In contrast, comprehensive physical training and walking aerobic exercise elicited improvements primarily in cognitive flexibility and inhibitory control, likely due to their rhythmic or multi-component movement patterns that produce relatively stable activation of prefrontal regulatory networks (Venegas-Sanabria et al., 2022; Ross et al., 2021). Taken together, the evidence supports physiologically and cognitively interpretable pathways linking exercise dose, modality and executive-function dimensions, providing a mechanistic foundation for the development of more precise exercise-based cognitive prescriptions.

### Clinical implications for exercise prescription

The dose–response findings of this study provide a practical foundation for developing precision exercise prescriptions for cognitive intervention. First, all three executive-function dimensions demonstrated stable improvements within the moderate cumulative dose range of approximately 300–1,000 METs⋅min/week, suggesting this interval as a feasible and safe core prescription target for both clinical and community-based programmes. Second, the optimal dose differed across dimensions: inhibitory control reached its peak at lower doses, whereas working memory and cognitive flexibility required higher cumulative doses to maximise improvement. These distinctions indicate that exercise prescriptions may be optimised through dimension-specific dose matching based on an individual’s primary cognitive deficits. Furthermore, the modality–dose combinations revealed that exercise joint cognitive training, comprehensive physical training and walking aerobic exercise consistently produced improvements within their respective effective dose windows. This finding highlights the importance of avoiding the assumption that all exercise modalities exert uniform effects. Instead, clinicians should consider modality selection and dose range as an integrated decision-making framework when designing interventions. From a functional perspective, improvements in working memory, cognitive flexibility and inhibitory control may also support the preservation of complex everyday behaviours that depend on planning, adaptive decision-making and behavioural regulation. Therefore, targeting executive function through appropriately dosed exercise may have implications beyond cognitive test performance, potentially contributing to the maintenance of daily functional independence in older adults with MCI. Overall, this study provides a dose-informed prescription model for older adults, individuals with mild cognitive impairment and those with executive-function deficits, supporting more precise, adherent and scalable non-pharmacological cognitive interventions in clinical and public health settings.

### Limitations and future directions

1. Heterogeneity in exercise-dose reporting

Inconsistencies in reporting exercise duration, frequency and intensity required dose standardisation using MET-based calculations in several studies, which may introduce estimation error and affect the precision of the dose–response curves.

2. Variability in executive-function assessment tools

Executive-function domains were assessed using diverse instruments (*e.g.*, Stroop, Trail Making, N-back, Flanker). Differences in sensitivity and task demands could contribute to variability in dose peaks across dimensions.

3. Uneven distribution of data across exercise modalities

Some modalities-such as mind–body exercise and resistance training-were represented by limited dose gradients, reducing the stability of modality–dose models and producing wide credible intervals, particularly in higher-dose regions.

4. Model dependence, effect modifiers and limited data in the high-dose range

Although Bayesian non-linear modelling enables flexible estimation of complex dose–response patterns, the results remain dependent on prior specifications and the density of dose observations. Mean age was examined as a study-level effect modifier using Bayesian network meta-regression. However, sex was not incorporated as an additional effect modifier in the primary analysis, and residual effect modification associated with sex therefore cannot be fully excluded. Furthermore, the use of aggregate study-level rather than individual-participant data limits the ability to examine participant-level interactions. Sparse observations at higher exercise doses also reduce the stability and interpretability of estimates in this range.

5. Directions for future research

Future trials should adopt standardised approaches for documenting exercise dose, employ consistent definitions of exercise modalities and incorporate wider dose gradients to more fully characterise non-linear effects. The use of standardised executive-function assessments, combined with multimodal neurophysiological measures-including fNIRS, fMRI and metabolic markers-would help to validate the underlying mechanisms of modality-specific effects and enhance the precision and generalisability of exercise-based cognitive prescriptions.

## Conclusion

Using a Bayesian dose–response modelling framework, this study delineated distinct response patterns linking exercise dose to the three core dimensions of executive function in older adults with mild cognitive impairment. The analysis identified dimension-specific optimal dose ranges and clarified the non-linear trajectories of cognitive flexibility, working memory and inhibitory control. In contrast to prior evidence that primarily reported whether exercise was effective, this study simultaneously quantified the peak dose ranges, differentiated the dose sensitivities across cognitive dimensions and mapped significant modality–dose combinations within a unified analytical system. These findings advance exercise-based cognitive intervention from isolated effect estimates to a quantifiable dose–prescription framework. By integrating dose information across modalities, the study further identified exercise joint cognitive training, comprehensive physical training and walking aerobic exercise as modalities demonstrating consistent advantages within defined dose windows, providing an empirical basis for modality selection. Collectively, this work advances the field from experience-based recommendations toward evidence-informed precision prescriptions. It offers a structured pathway for non-pharmacological cognitive interventions targeting older adults with MCI and those with executive-function deficits, and establishes a methodological foundation for future development of scalable, cross-modality and dimension-specific exercise prescriptions.

##  Supplemental Information

10.7717/peerj.21805/supp-1 Supplemental Information 1 PRISMA Checklist

10.7717/peerj.21805/supp-2 Supplemental Information 2 Retrieval strategies

10.7717/peerj.21805/supp-3 Figure S1 Network plot of overall exercise dose comparisons for cognitive flexibility, illustrating the number of studies and direct comparisons across dose nodes

10.7717/peerj.21805/supp-4 Supplemental Information 4 Network plot of overall exercise dose comparisons for working memory, showing the distribution of studies and connections among dose nodes

10.7717/peerj.21805/supp-5 Figure S3 Network plot of overall exercise dose comparisons for inhibitory control, depicting study contributions and direct links between dose nodes

10.7717/peerj.21805/supp-6 Figure S4 Network plot of exercise modality–dose combinations for cognitive flexibility, showing the available direct comparisons and study connections among modalities and dose levels

10.7717/peerj.21805/supp-7 Figure S5 Network plot of exercise modality–dose combinations for working memory, illustrating study contributions and direct links across modalities and dose levels

10.7717/peerj.21805/supp-8 Figure S6 Network plot of exercise modality–dose combinations for inhibitory control, depicting the distribution of studies and the direct comparisons between modality–dose nodes

10.7717/peerj.21805/supp-9 Supplemental Information 9 Intended audience

## Additional Information and Declarations

### Data Availability

The following information was supplied regarding data availability: This is a Systematic review/Meta-analysis.
