# Supplementary material for: Optimizing exercise prescriptions for older adults with mild cognitive impairment: a dose–response network meta-analysis of modality–dose combinations on executive function
Source: PeerJ. 2026 Oct 20;14:e21805. doi: 10.7717/peerj.21805 (PMC13693602; doi:10.7717/peerj.21805)
Supplement: Supplementary file 2 [file peerj-14-21805-s004.pdf]

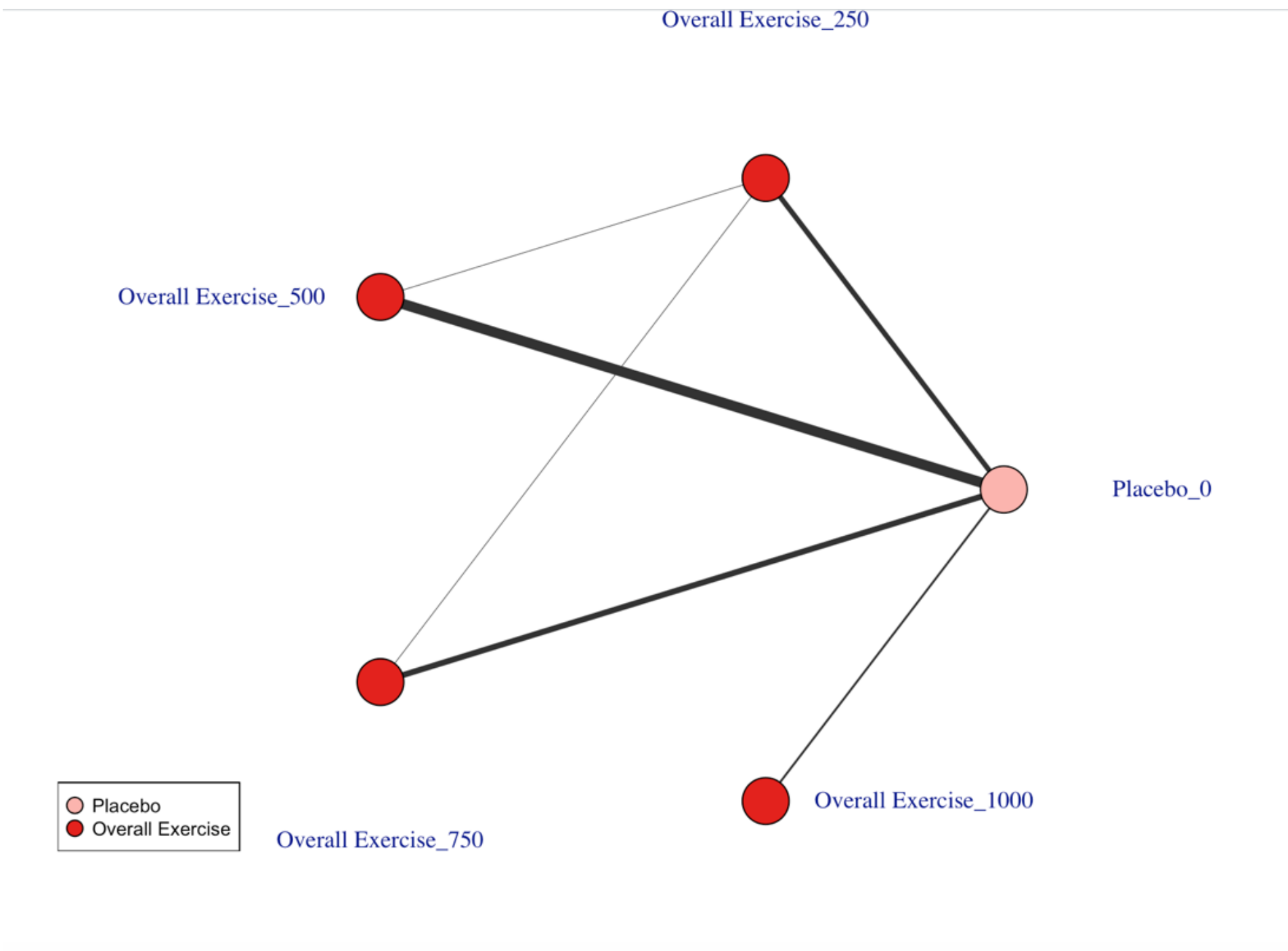

Figure S2. Network plot of overall exercise dose comparisons for working memory, showing the distribution of studies and connections among dose nodes.
