# Supplementary material for: Optimizing exercise prescriptions for older adults with mild cognitive impairment: a dose–response network meta-analysis of modality–dose combinations on executive function
Source: PeerJ. 2026 Oct 20;14:e21805. doi: 10.7717/peerj.21805 (PMC13693602; doi:10.7717/peerj.21805)
Supplement: Supplementary file 3 [file peerj-14-21805-s005.pdf]

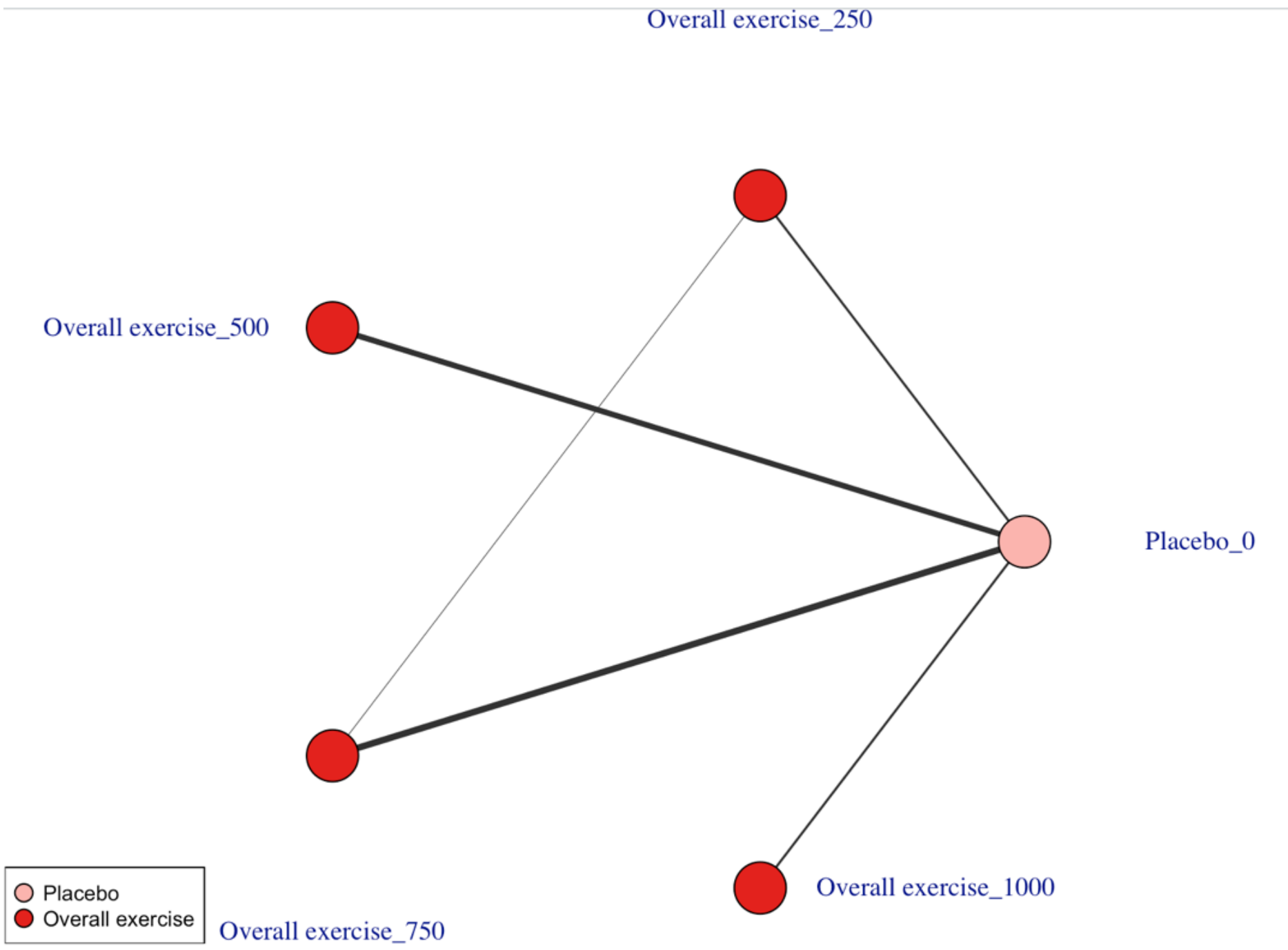
Overall exercise_250
Overall exercise_500
Placebo_0
Placebo
Overall exercise
Overall exercise_750
Overall exercise_1000
