# Supplementary material for: Optimizing exercise prescriptions for older adults with mild cognitive impairment: a dose–response network meta-analysis of modality–dose combinations on executive function
Source: PeerJ. 2026 Oct 20;14:e21805. doi: 10.7717/peerj.21805 (PMC13693602; doi:10.7717/peerj.21805)
Supplement: Supplementary file 4 [file peerj-14-21805-s006.pdf]

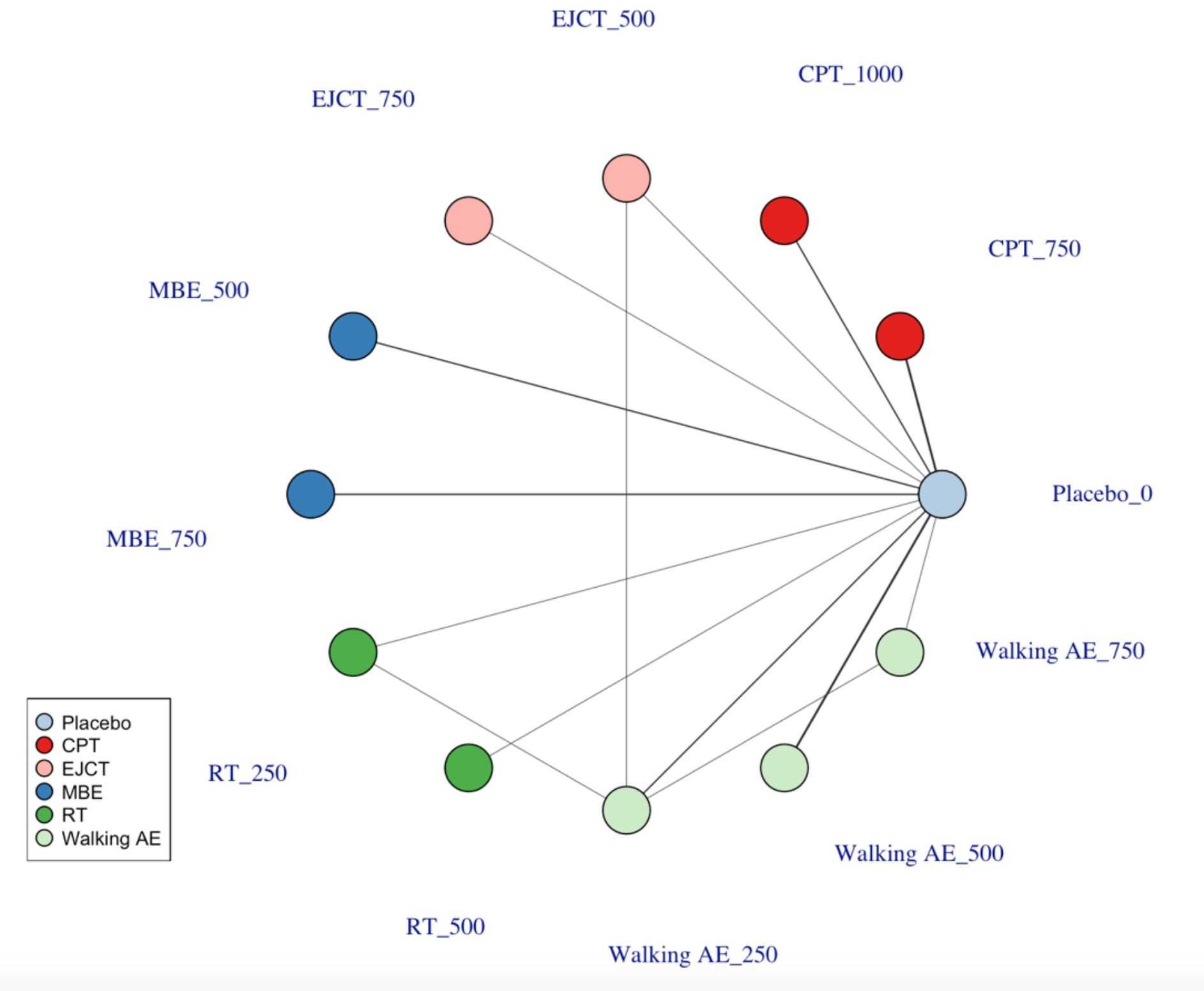
EJCT_500
CPT_1000
EJCT_750
CPT_750
MBE_500
Placebo_0
MBE_750
Walking AE_750
Placebo
CPT
EJCT
MBE
RT
Walking AE
RT_250
Walking AE_500
RT_500
Walking AE_250
