# Supplementary material for: Optimizing exercise prescriptions for older adults with mild cognitive impairment: a dose–response network meta-analysis of modality–dose combinations on executive function
Source: PeerJ. 2026 Oct 20;14:e21805. doi: 10.7717/peerj.21805 (PMC13693602; doi:10.7717/peerj.21805)
Supplement: Supplementary file 5 [file peerj-14-21805-s007.pdf]

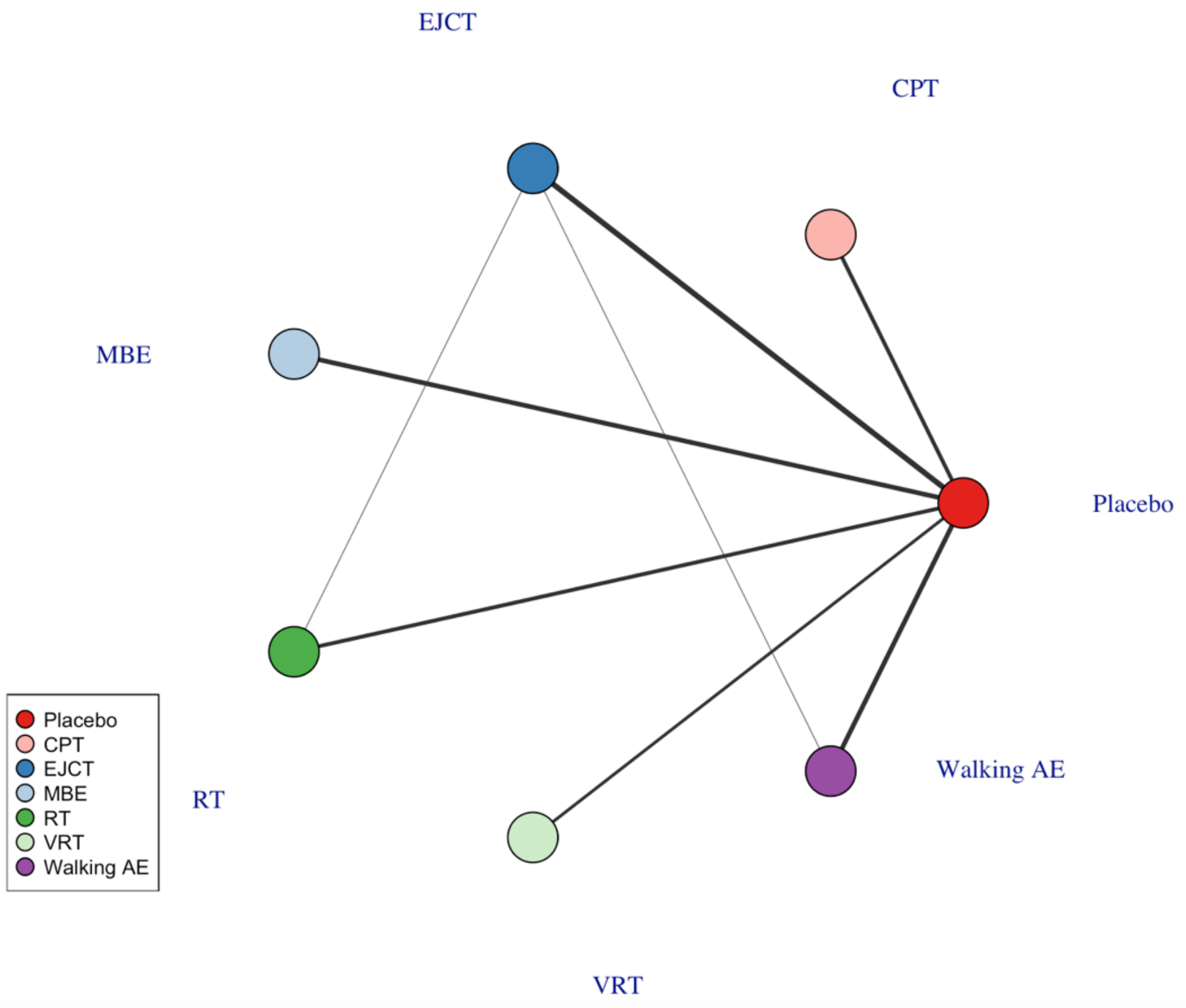
EJCT
CPT
MBE
Placebo
RT
Walking AE
VRT
Placebo
CPT
EJCT
MBE
RT
VRT
Walking AE
