# Supplementary material for: Optimizing exercise prescriptions for older adults with mild cognitive impairment: a dose–response network meta-analysis of modality–dose combinations on executive function
Source: PeerJ. 2026 Oct 20;14:e21805. doi: 10.7717/peerj.21805 (PMC13693602; doi:10.7717/peerj.21805)
Supplement: Supplementary file 8 [file peerj-14-21805-s002.docx]

**Retrieval Strategies:(20271)**

**Cochrane Library (6068)**

**#1 Population**: (MCI OR cognitive disorder OR mental deterioration OR cognitive decline OR Mild Cognitive Impairment OR cognitive impairment OR cognitive dysfunction):ti

**#2 Intervention**: (water sports OR tai chi OR yoga OR movement OR run OR isometric OR dance OR martial art OR swim OR core control OR resistance OR mind-body exercise OR endurance OR activity OR stretch OR motor control OR sport OR Sprint OR physical OR activities OR kinesiotherapy OR virtual reality OR walk OR high intensity interval OR strength OR hydrotherapy OR aerobic OR exercise OR train OR functional training OR athletic OR pilates OR sports combined training OR activity):ti, ab, kw

**#3 Outcomes**: (cognitive flexibility OR executive OR inhibition OR switch OR memory OR cognitive):ti, ab, kw

**Search algorithm**: Trials matching "#4 - #1 AND #2 AND #3"

**Web of Science (11987)**

**#1 Population**: AB (MCI OR cognitive disorder OR mental deterioration OR cognitive decline OR Mild Cognitive Impairment OR cognitive impairment OR cognitive dysfunction)

**#2 Intervention**: AB (water sports OR tai chi OR yoga OR movement OR run OR isometric OR dance OR martial art OR swim OR core control OR resistance OR mind-body exercise OR endurance OR activity OR stretch OR motor control OR sport OR Sprint OR physical OR activities OR kinesiotherapy OR virtual reality OR walk OR high intensity interval OR strength OR hydrotherapy OR aerobic OR exercise OR train OR functional training OR athletic OR pilates OR sports combined training OR activity)

**#3 Outcomes**: AB (cognitive flexibility OR executive OR inhibition OR switch OR memory OR cognitive)

**Search algorithm**: #3 AND #2 AND #1 and Article (Document Types)

**PubMed (681)**

**#1 Population:** MCI[Title] OR cognitive disorder[Title] OR mental deterioration[Title] OR cognitive decline[Title] OR Mild Cognitive Impairment[Title] OR cognitive impairment[Title] OR cognitive dysfunction[Title]

**#2 Intervention:** water sports[Title/Abstract] OR tai chi[Title/Abstract] OR yoga[Title/Abstract] OR movement[Title/Abstract] OR run[Title/Abstract] OR isometric[Title/Abstract] OR dance[Title/Abstract] OR martial art[Title/Abstract] OR swim[Title/Abstract] OR core control[Title/Abstract] OR resistance[Title/Abstract] OR mind-body exercise[Title/Abstract] OR endurance[Title/Abstract] OR activity[Title/Abstract] OR stretch[Title/Abstract] OR motor control[Title/Abstract] OR sport[Title/Abstract] OR Sprint[Title/Abstract] OR physical[Title/Abstract] OR activities[Title/Abstract] OR kinesiotherapy[Title/Abstract] OR virtual reality[Title/Abstract] OR walk[Title/Abstract] OR high intensity interval[Title/Abstract] OR strength[Title/Abstract] OR hydrotherapy[Title/Abstract] OR aerobic[Title/Abstract] OR exercise[Title/Abstract] OR train[Title/Abstract] OR functional training[Title/Abstract] OR athletic[Title/Abstract] OR pilates[Title/Abstract] OR sports combined training[Title/Abstract] OR activity[Title/Abstract]

**#3 Outcomes:** cognitive flexibility[Title/Abstract] OR executive[Title/Abstract] OR inhibition[Title/Abstract] OR switch[Title/Abstract] OR memory[Title/Abstract] OR cognitive[Title/Abstract]

**Search algorithm**: #1 AND #2 AND #3 **Filters**: Clinical Trial

**Embase (1535)**

#1 Population: mci:ti OR 'cognitive disorder':ti OR 'mental deterioration':ti OR 'cognitive decline':ti OR 'mild cognitive impairment':ti OR 'cognitive impairment':ti OR 'cognitive dysfunction':ti

**#2 Intervention**: 'water sports':ab OR 'tai chi':ab OR yoga:ab OR movement:ab OR run:ab OR isometric:ab OR dance:ab OR 'martial art':ab OR swim:ab OR 'core control':ab OR resistance:ab OR 'mind-body exercise':ab OR endurance:ab OR stretch:ab OR 'motor control':ab OR sport:ab OR sprint:ab OR physical:ab OR activities:ab OR kinesiotherapy:ab OR 'virtual reality':ab OR walk:ab OR 'high intensity interval':ab OR strength:ab OR hydrotherapy:ab OR aerobic:ab OR exercise:ab OR train:ab OR 'functional training':ab OR athletic:ab OR pilates:ab OR 'sports combined training':ab OR activity:ab

**#3 Outcomes**: 'cognitive flexibility':ab OR executive:ab OR inhibition:ab OR switch:ab OR memory:ab OR cognitive:ab

**Search algorithm**: #1 AND #2 AND #3 AND ('randomized controlled trial'/de)

Table S1. Pairwise comparisons of overall exercise dose effects on cognitive flexibility (MBNMA estimates, direct and indirect evidence).

| Comparison | p-value | Median | 0.025 | 0.975 |
| --- | --- | --- | --- | --- |
| Overall exercise_1500 vs Overall exercise_750 | 0.375 |  |  |  |
| → direct |  | -0.029 | -0.472 | 0.335 |
| → indirect |  | 0.083 | -0.022 | 0.185 |
| → MBNMA |  | 0.081 | -0.023 | 0.181 |
|  |  |  |  |  |
| Overall exercise_1000 vs Overall exercise_750 | 0.112 |  |  |  |
| → direct |  | 0.024 | -0.591 | 0.661 |
| → indirect |  | 0.027 | -0.009 | 0.064 |
| → MBNMA |  | 0.027 | -0.008 | 0.06 |
|  |  |  |  |  |
| Overall exercise_500 vs Placebo_0 | 0.432 |  |  |  |
| → direct |  | 0.104 | -0.026 | 0.236 |
| → indirect |  | 0.028 | -0.048 | 0.103 |
| → MBNMA |  | 0.054 | -0.015 | 0.121 |
|  |  |  |  |  |
| Overall exercise_250 vs Placebo_0 | 0.422 |  |  |  |
| → direct |  | 0.044 | -0.1 | 0.173 |
| → indirect |  | 0.026 | -0.009 | 0.062 |
| → MBNMA |  | 0.027 | -0.008 | 0.06 |

Table S2. Pairwise comparisons of overall exercise dose effects on working memory (MBNMA estimates, direct and indirect evidence).

| Comparison | p-value | Median | 0.025 | 0.975 |
| --- | --- | --- | --- | --- |
| Overall Exercise_500 vs Overall Exercise_250 | 0.131 |  |  |  |
| → direct |  | -0.022 | -0.757 | 0.596 |
| → indirect |  | 0.021 | -0.014 | 0.058 |
| → MBNMA |  | 0.025 | -0.013 | 0.058 |
|  |  |  |  |  |
| Overall Exercise_1000 vs Placebo_0 | 0.565 |  |  |  |
| → direct |  | 0.13 | -0.222 | 0.516 |
| → indirect |  | 0.063 | -0.081 | 0.221 |
| → MBNMA |  | 0.099 | -0.053 | 0.231 |
|  |  |  |  |  |
| Overall Exercise_750 vs Placebo_0 | 0.823 |  |  |  |
| → direct |  | 0.046 | -0.117 | 0.215 |
| → indirect |  | 0.082 | -0.062 | 0.235 |
| → MBNMA |  | 0.074 | -0.039 | 0.174 |

Table S3. Pairwise comparisons of overall exercise dose effects on inhibitory control (MBNMA estimates, direct and indirect evidence).

| Comparison | p-value | Median | 0.025 | 0.975 |
| --- | --- | --- | --- | --- |
| Overall exercise_1000 vs Placebo_0 | 0.497 |  |  |  |
| → direct |  | 0.06 | -0.364 | 0.492 |
| → indirect |  | 0.289 | -0.009 | 0.563 |
| → MBNMA |  | 0.222 | -0.007 | 0.454 |
|  |  |  |  |  |
| Overall exercise_750 vs Placebo_0 | 0.552 |  |  |  |
| → direct |  | 0.24 | -0.004 | 0.483 |
| → indirect |  | 0.097 | -0.149 | 0.335 |
| → MBNMA |  | 0.166 | -0.005 | 0.34 |
|  |  |  |  |  |
| Overall exercise_500 vs Placebo_0 | 0.7 |  |  |  |
| → direct |  | 0.097 | -0.159 | 0.345 |
| → indirect |  | 0.114 | -0.02 | 0.248 |
| → MBNMA |  | 0.111 | -0.004 | 0.227 |
|  |  |  |  |  |
| Overall exercise_250 vs Placebo_0 | 0.236 |  |  |  |
| → direct |  | 0.098 | -0.409 | 0.553 |
| → indirect |  | 0.056 | -0.005 | 0.11 |
| → MBNMA |  | 0.055 | -0.002 | 0.113 |

Table S4. Predicted dose–response effects of overall exercise on cognitive flexibility (MBNMA estimates).

| agent | dose | mean | sd | 2.5% | 25% | 50% | 75% | 97.5% |
| --- | --- | --- | --- | --- | --- | --- | --- | --- |
| Placebo | 0 | 0.1668173 | 0.1049941 | 0.023514311 | 0.08603853 | 0.1468027 | 0.2268143 | 0.4152294 |
| Overall exercise | 0 | 0.1668173 | 0.1049941 | 0.023514311 | 0.08603853 | 0.1468027 | 0.2268143 | 0.4152294 |
| Overall exercise | 170 | 0.1986426 | 0.1076222 | 0.046262341 | 0.11676517 | 0.1790082 | 0.2607531 | 0.4523652 |
| Overall exercise | 330 | 0.2227041 | 0.1114307 | 0.060007407 | 0.13948484 | 0.2030361 | 0.2881014 | 0.4833145 |
| Overall exercise | 500 | 0.2420094 | 0.1149751 | 0.067107889 | 0.15834975 | 0.2231257 | 0.3111208 | 0.5086464 |
| Overall exercise | 670 | 0.254865 | 0.1176895 | 0.072845573 | 0.17087419 | 0.2357097 | 0.324198 | 0.5239874 |
| Overall exercise | 830 | 0.2610725 | 0.1204882 | 0.069608504 | 0.17584279 | 0.2434087 | 0.3330224 | 0.5269563 |
| Overall exercise | 1000 | 0.261408 | 0.1259941 | 0.05128009 | 0.17407266 | 0.2478134 | 0.3370566 | 0.5338716 |
| Overall exercise | 1200 | 0.2535453 | 0.1400299 | 0.003719679 | 0.15606813 | 0.2428188 | 0.3379223 | 0.5556255 |
| Overall exercise | 1300 | 0.2462664 | 0.1515196 | -0.036133467 | 0.14049578 | 0.2367893 | 0.340343 | 0.5676146 |
| Overall exercise | 1500 | 0.2250132 | 0.1851692 | -0.123255065 | 0.0988863 | 0.2201492 | 0.3469474 | 0.6120498 |

Table S5. Predicted dose–response effects of overall exercise on working memory (MBNMA estimates).

| Agent | Dose | Mean | SD | **2.5%** | **25%** | **50%** | **75%** | **97.5%** |
| --- | --- | --- | --- | --- | --- | --- | --- | --- |
| Placebo | 0 | 0.1658104 | 0.1021536 | 0.02292046 | 0.08587906 | 0.1503764 | 0.2269874 | 0.4107322 |
| Overall Exercise | 0 | 0.1658104 | 0.1021536 | 0.02292046 | 0.08587906 | 0.1503764 | 0.2269874 | 0.4107322 |
| Overall Exercise | 110 | 0.177768 | 0.1052103 | 0.02316088 | 0.09813813 | 0.1637869 | 0.2418883 | 0.4254949 |
| Overall Exercise | 220 | 0.1885527 | 0.1104037 | 0.017217 | 0.10762571 | 0.1747578 | 0.2557122 | 0.4413657 |
| Overall Exercise | 330 | 0.1981645 | 0.1140355 | 0.01489767 | 0.11581564 | 0.1855279 | 0.2678435 | 0.4544502 |
| Overall Exercise | 440 | 0.2066033 | 0.1150077 | 0.02080915 | 0.12276668 | 0.1938086 | 0.276621 | 0.4654359 |
| Overall Exercise | 560 | 0.2144717 | 0.1142798 | 0.02865239 | 0.13126833 | 0.2018877 | 0.2832223 | 0.473381 |
| Overall Exercise | 670 | 0.2204582 | 0.1154503 | 0.03187929 | 0.1364796 | 0.209469 | 0.2900685 | 0.4808412 |
| Overall Exercise | 780 | 0.2252717 | 0.1236681 | 0.01655669 | 0.13835344 | 0.2137564 | 0.2999445 | 0.4961008 |
| Overall Exercise | 890 | 0.2289124 | 0.143957 | -0.02607259 | 0.12938241 | 0.2201881 | 0.3211547 | 0.5352081 |
| Overall Exercise | 1000 | 0.2313801 | 0.1785766 | -0.09667386 | 0.10903526 | 0.2256913 | 0.3485884 | 0.6008054 |

Table S6. Predicted dose–response effects of overall exercise on inhibitory control (MBNMA estimates).

| agent | dose | mean | sd | **2.5%** | **25%** | **50%** | **75%** | **97.5%** |
| --- | --- | --- | --- | --- | --- | --- | --- | --- |
| Placebo | 0 | 0.1689994 | 0.1051675 | 0.02362238 | 0.08739085 | 0.1495584 | 0.2278507 | 0.4273247 |
| Overall exercise | 0 | 0.1689994 | 0.1051675 | 0.02362238 | 0.08739085 | 0.1495584 | 0.2278507 | 0.4273247 |
| Overall exercise | 110 | 0.2175846 | 0.1166194 | 0.03497333 | 0.12909586 | 0.2036966 | 0.2881887 | 0.4875757 |
| Overall exercise | 220 | 0.2579993 | 0.1344041 | 0.02815767 | 0.16108493 | 0.2492217 | 0.3418018 | 0.5569299 |
| Overall exercise | 330 | 0.2902433 | 0.1469131 | 0.0315402 | 0.18652819 | 0.2821212 | 0.3843152 | 0.6030333 |
| Overall exercise | 440 | 0.3143168 | 0.1507876 | 0.04585896 | 0.21015877 | 0.3069101 | 0.4122663 | 0.6311261 |
| Overall exercise | 560 | 0.3312603 | 0.146187 | 0.0757678 | 0.22743991 | 0.3240035 | 0.4259638 | 0.6414006 |
| Overall exercise | 670 | 0.3382498 | 0.1391997 | 0.09684615 | 0.24291604 | 0.3275737 | 0.4244784 | 0.6329407 |
| Overall exercise | 780 | 0.3370687 | 0.1406858 | 0.0856075 | 0.24199568 | 0.3278316 | 0.4239168 | 0.6354388 |
| Overall exercise | 890 | 0.3277171 | 0.1647079 | 0.01365096 | 0.21757807 | 0.3240546 | 0.4310313 | 0.6657869 |
| Overall exercise | 1000 | 0.3101948 | 0.2175894 | -0.1190276 | 0.1732742 | 0.3062701 | 0.4480979 | 0.757613 |

Table S7. Pairwise comparisons of exercise modality–dose combinations on cognitive flexibility (MBNMA estimates, direct and indirect evidence).

| Comparison | p-value | Type | Median | 0.025 | 0.975 |
| --- | --- | --- | --- | --- | --- |
| Walking AE_250 vs RT_250 | 0.573 | direct | 0.003 | -0.806 | 0.782 |
|  |  | indirect | 0.26 | -0.158 | 0.671 |
|  |  | MBNMA | 0.212 | -0.129 | 0.551 |
| Walking AE_250 vs EJCT_500 | 0.649 | direct | -0.112 | -0.975 | 0.758 |
|  |  | indirect | 0.155 | -0.384 | 0.713 |
|  |  | MBNMA | 0.085 | -0.344 | 0.54 |
| Walking AE_750 vs Placebo_0 | 0.776 | direct | 0.323 | -0.519 | 1.254 |
|  |  | indirect | 0.401 | -0.204 | 1.024 |
|  |  | MBNMA | 0.341 | -0.172 | 0.859 |
| Walking AE_500 vs Placebo_0 | 0.859 | direct | 0.252 | -0.162 | 0.688 |
|  |  | indirect | 0.186 | -0.372 | 0.749 |
|  |  | MBNMA | 0.227 | -0.115 | 0.573 |
| RT_500 vs Placebo_0 | 0.544 | direct | -0.339 | -1.11 | 0.415 |
|  |  | indirect | 0.227 | -1 | 1.504 |
|  |  | MBNMA | -0.195 | -0.791 | 0.422 |
| MBE_750 vs Placebo_0 | 0.789 | direct | 0.116 | -0.443 | 0.656 |
|  |  | indirect | 0.195 | -0.688 | 0.992 |
|  |  | MBNMA | 0.133 | -0.29 | 0.585 |
| MBE_500 vs Placebo_0 | 0.8 | direct | 0.128 | -0.409 | 0.674 |
|  |  | indirect | 0.067 | -0.308 | 0.436 |
|  |  | MBNMA | 0.088 | -0.193 | 0.39 |
| EJCT_750 vs Placebo_0 | 0.762 | direct | -0.107 | -1.052 | 0.854 |
|  |  | indirect | 0.166 | -0.758 | 1.078 |
|  |  | MBNMA | 0.045 | -0.643 | 0.696 |
| CPT_1000 vs Placebo_0 | 0.291 | direct | 0.073 | -0.472 | 0.634 |
|  |  | indirect | 0.636 | 0.028 | 1.157 |
|  |  | MBNMA | 0.363 | -0.076 | 0.744 |
| CPT_750 vs Placebo_0 | 0.331 | direct | 0.461 | 0.038 | 0.856 |
|  |  | indirect | 0.061 | -0.389 | 0.48 |
|  |  | MBNMA | 0.273 | -0.057 | 0.558 |

Table S8. Pairwise comparisons of exercise modality–dose combinations on working memory (MBNMA estimates, direct and indirect evidence).

| Comparison | p-value | Median | 0.025 | 0.975 |
| --- | --- | --- | --- | --- |
| Walking AE_250 vs EJCT_500 | 0.378 |  |  |  |
| → direct |  | 0.076 | –0.590 | 0.803 |
| → indirect |  | –0.056 | –0.219 | 0.104 |
| → MBNMA |  | –0.045 | –0.193 | 0.122 |
| Walking AE_500 vs Placebo_0 | 0.31 |  |  |  |
| → direct |  | 0.014 | –0.167 | 0.199 |
| → indirect |  | 0.144 | –0.973 | 1.138 |
| → MBNMA |  | 0.029 | –0.154 | 0.201 |
| VRT_1000 vs Placebo_0 | 0.675 |  |  |  |
| → direct |  | 0.335 | –0.276 | 0.982 |
| → indirect |  | 0.048 | –0.662 | 0.699 |
| → MBNMA |  | 0.17 | –0.264 | 0.646 |
| VRT_750 vs Placebo_0 | 0.595 |  |  |  |
| → direct |  | –0.035 | –0.648 | 0.508 |
| → indirect |  | 0.238 | –0.195 | 0.672 |
| → MBNMA |  | 0.127 | –0.198 | 0.484 |
| VRT_500 vs Placebo_0 | 0.572 |  |  |  |
| → direct |  | 0.199 | –0.420 | 0.772 |
| → indirect |  | 0.081 | –0.198 | 0.351 |
| → MBNMA |  | 0.085 | –0.132 | 0.323 |
| RT_500 vs Placebo_0 | 0.441 |  |  |  |
| → direct |  | 0.1 | –0.176 | 0.39 |
| → indirect |  | 0.219 | –0.675 | 1.171 |
| → MBNMA |  | 0.115 | –0.135 | 0.459 |
| RT_250 vs Placebo_0 | 0.479 |  |  |  |
| → direct |  | 0.086 | –0.366 | 0.535 |
| → indirect |  | 0.063 | –0.075 | 0.192 |
| → MBNMA |  | 0.057 | –0.068 | 0.229 |
| MBE_750 vs Placebo_0 | 0.691 |  |  |  |
| → direct |  | 0.081 | –0.248 | 0.465 |
| → indirect |  | –0.057 | –0.493 | 0.396 |
| → MBNMA |  | 0.029 | –0.213 | 0.296 |
| MBE_500 vs Placebo_0 | 0.595 |  |  |  |
| → direct |  | –0.056 | –0.405 | 0.313 |
| → indirect |  | 0.056 | –0.141 | 0.237 |
| → MBNMA |  | 0.019 | –0.142 | 0.197 |
| MBE_250 vs Placebo_0 | 0.672 |  |  |  |
| → direct |  | 0.013 | –0.203 | 0.215 |
| → indirect |  | 0.015 | –0.088 | 0.114 |
| → MBNMA |  | 0.01 | –0.071 | 0.099 |
| EJCT_750 vs Placebo_0 | 0.466 |  |  |  |
| → direct |  | 0.022 | –0.230 | 0.267 |
| → indirect |  | 0.257 | –0.129 | 0.637 |
| → MBNMA |  | 0.084 | –0.114 | 0.295 |
| EJCT_250 vs Placebo_0 | 0.272 |  |  |  |
| → direct |  | 0.003 | –0.405 | 0.504 |
| → indirect |  | 0.027 | –0.040 | 0.096 |
| → MBNMA |  | 0.028 | –0.038 | 0.098 |
| CPT_1000 vs Placebo_0 | 0.958 |  |  |  |
| → direct |  | 0.023 | –0.388 | 0.451 |
| → indirect |  | 0.03 | –0.373 | 0.496 |
| → MBNMA |  | 0.039 | –0.223 | 0.319 |
| CPT_750 vs Placebo_0 | 0.822 |  |  |  |
| → direct |  | 0.023 | –0.368 | 0.416 |
| → indirect |  | 0.022 | –0.225 | 0.297 |
| → MBNMA |  | 0.03 | –0.167 | 0.239 |
| CPT_500 vs Placebo_0 | 0.747 |  |  |  |
| → direct |  | 0.006 | –0.266 | 0.293 |
| → indirect |  | 0.033 | –0.138 | 0.206 |
| → MBNMA |  | 0.02 | –0.112 | 0.159 |

Table S9. Pairwise comparisons of exercise modality–dose combinations on inhibitory control (MBNMA estimates, direct and indirect evidence).

| Comparison | p-value | Median | 0.025 | 0.975 |
| --- | --- | --- | --- | --- |
| Walking AE_250 vs RT_250 | 0.585 |  |  |  |
| → direct |  | 0.024 | -0.732 | 0.776 |
| → indirect |  | 0.267 | -0.137 | 0.672 |
| → MBNMA |  | 0.217 | -0.119 | 0.546 |
|  |  |  |  |  |
| Walking AE_250 vs EJCT_500 | 0.64 |  |  |  |
| → direct |  | -0.116 | -1.026 | 0.771 |
| → indirect |  | 0.166 | -0.345 | 0.726 |
| → MBNMA |  | 0.094 | -0.36 | 0.551 |
|  |  |  |  |  |
| Walking AE_750 vs Placebo_0 | 0.804 |  |  |  |
| → direct |  | 0.321 | -0.484 | 1.211 |
| → indirect |  | 0.394 | -0.176 | 0.995 |
| → MBNMA |  | 0.342 | -0.147 | 0.857 |
|  |  |  |  |  |
| Walking AE_500 vs Placebo_0 | 0.848 |  |  |  |
| → direct |  | 0.252 | -0.153 | 0.711 |
| → indirect |  | 0.193 | -0.373 | 0.706 |
| → MBNMA |  | 0.228 | -0.098 | 0.571 |
|  |  |  |  |  |
| RT_500 vs Placebo_0 | 0.533 |  |  |  |
| → direct |  | -0.344 | -1.15 | 0.391 |
| → indirect |  | 0.197 | -1.045 | 1.421 |
| → MBNMA |  | -0.196 | -0.835 | 0.425 |
|  |  |  |  |  |
| MBE_750 vs Placebo_0 | 0.81 |  |  |  |
| → direct |  | 0.119 | -0.458 | 0.707 |
| → indirect |  | 0.183 | -0.639 | 1.019 |
| → MBNMA |  | 0.135 | -0.307 | 0.59 |
|  |  |  |  |  |
| MBE_500 vs Placebo_0 | 0.807 |  |  |  |
| → direct |  | 0.12 | -0.401 | 0.697 |
| → indirect |  | 0.081 | -0.294 | 0.449 |
| → MBNMA |  | 0.09 | -0.205 | 0.394 |
|  |  |  |  |  |
| EJCT_750 vs Placebo_0 | 0.767 |  |  |  |
| → direct |  | -0.116 | -1.099 | 0.892 |
| → indirect |  | 0.158 | -0.771 | 1.069 |
| → MBNMA |  | 0.024 | -0.618 | 0.683 |
|  |  |  |  |  |
| CPT_1000 vs Placebo_0 | 0.314 |  |  |  |
| → direct |  | 0.084 | -0.475 | 0.621 |
| → indirect |  | 0.636 | 0.072 | 1.164 |
| → MBNMA |  | 0.351 | -0.074 | 0.78 |
|  |  |  |  |  |
| CPT_750 vs Placebo_0 | 0.303 |  |  |  |
| → direct |  | 0.469 | 0.033 | 0.893 |
| → indirect |  | 0.046 | -0.383 | 0.455 |
| → MBNMA |  | 0.263 | -0.055 | 0.585 |

Table S10. Predicted effects of exercise modality–dose combinations on cognitive flexibility (MBNMA estimates).

| agent | dose | mean | sd | 0.025 | 0.25 | 0.5 | 0.75 | 0.975 |
| --- | --- | --- | --- | --- | --- | --- | --- | --- |
| Placebo | 0 | 0.168876176 | 0.1063261 | 0.018734399 | 0.087919555 | 0.15001639 | 0.230073 | 0.4185282 |
| CPT | 0 | 0.168876176 | 0.1063261 | 0.018734399 | 0.087919555 | 0.15001639 | 0.230073 | 0.4185282 |
| CPT | 110 | 0.384502999 | 0.1903069 | 0.015286402 | 0.260751393 | 0.38065257 | 0.5043217 | 0.7781868 |
| CPT | 220 | 0.548965033 | 0.2927739 | -0.044652854 | 0.368723854 | 0.54945364 | 0.7404717 | 1.1161586 |
| CPT | 330 | 0.662262279 | 0.3595489 | -0.071905889 | 0.445286097 | 0.66301858 | 0.8960847 | 1.355461 |
| CPT | 440 | 0.724394737 | 0.3856258 | -0.07722341 | 0.490801781 | 0.72137521 | 0.9757489 | 1.4663464 |
| CPT | 560 | 0.73382237 | 0.368582 | -0.016102454 | 0.506955668 | 0.73317968 | 0.9751176 | 1.4447652 |
| CPT | 670 | 0.688973906 | 0.3169548 | 0.041179614 | 0.488575165 | 0.6891219 | 0.8977044 | 1.3042946 |
| CPT | 780 | 0.592960653 | 0.250534 | 0.090015204 | 0.43061391 | 0.59265401 | 0.7513014 | 1.0919486 |
| CPT | 890 | 0.445782613 | 0.2346967 | -0.018073343 | 0.292668466 | 0.44569348 | 0.5917831 | 0.9282367 |
| CPT | 1000 | 0.247439783 | 0.3493687 | -0.43393529 | 0.019590059 | 0.2444843 | 0.4645117 | 0.9592718 |
| EJCT | 0 | 0.168876176 | 0.1063261 | 0.018734399 | 0.087919555 | 0.15001639 | 0.230073 | 0.4185282 |
| EJCT | 83 | 0.257291991 | 0.2059999 | -0.145444318 | 0.116250638 | 0.25006853 | 0.3890414 | 0.6677908 |
| EJCT | 170 | 0.323610721 | 0.3254642 | -0.320630607 | 0.109964891 | 0.31887188 | 0.5358954 | 0.9697587 |
| EJCT | 250 | 0.360783857 | 0.3934765 | -0.414044857 | 0.100082789 | 0.35462871 | 0.6196994 | 1.1433004 |
| EJCT | 330 | 0.375145394 | 0.4183692 | -0.448505286 | 0.098106028 | 0.36329275 | 0.6504242 | 1.2102389 |
| EJCT | 420 | 0.364035136 | 0.3990543 | -0.4144963 | 0.094249843 | 0.35225054 | 0.6212288 | 1.1654846 |
| EJCT | 500 | 0.329922028 | 0.3523634 | -0.352656114 | 0.093413936 | 0.32228578 | 0.5608165 | 1.059104 |
| EJCT | 580 | 0.272997323 | 0.3124721 | -0.326151533 | 0.06283742 | 0.2721058 | 0.4779871 | 0.8769432 |
| EJCT | 670 | 0.181690041 | 0.3627786 | -0.526771536 | -0.059081609 | 0.18241173 | 0.4192852 | 0.8812444 |
| EJCT | 750 | 0.07629069 | 0.5320649 | -0.942672868 | -0.269572694 | 0.06379874 | 0.4306385 | 1.0964313 |
| MBE | 0 | 0.168876176 | 0.1063261 | 0.018734399 | 0.087919555 | 0.15001639 | 0.230073 | 0.4185282 |
| MBE | 83 | 0.205614484 | 0.1828092 | -0.13620677 | 0.083569716 | 0.20128835 | 0.318536 | 0.5906189 |
| MBE | 170 | 0.237634689 | 0.2795065 | -0.311502238 | 0.054388488 | 0.24143971 | 0.415327 | 0.794202 |
| MBE | 250 | 0.261217296 | 0.3388471 | -0.399420912 | 0.042195106 | 0.2654466 | 0.4724194 | 0.9374157 |
| MBE | 330 | 0.279184326 | 0.365443 | -0.422915353 | 0.042571285 | 0.28419795 | 0.5081175 | 1.0117525 |
| MBE | 420 | 0.292684864 | 0.3570301 | -0.385289389 | 0.062926048 | 0.29355476 | 0.5154312 | 1.0105255 |
| MBE | 500 | 0.298718791 | 0.3200045 | -0.31677657 | 0.088395984 | 0.30054071 | 0.4992947 | 0.9406653 |
| MBE | 580 | 0.29913714 | 0.2683119 | -0.221620367 | 0.11944761 | 0.30014046 | 0.4649765 | 0.8407752 |
| MBE | 670 | 0.292895413 | 0.2411592 | -0.176820932 | 0.140552297 | 0.28888987 | 0.4421162 | 0.7726544 |
| MBE | 750 | 0.28138066 | 0.3119095 | -0.33229602 | 0.086578046 | 0.27992396 | 0.4699737 | 0.8825796 |
| RT | 0 | 0.168876176 | 0.1063261 | 0.018734399 | 0.087919555 | 0.15001639 | 0.230073 | 0.4185282 |
| RT | 56 | 0.255374854 | 0.1759817 | -0.076870375 | 0.137648831 | 0.24783872 | 0.3672196 | 0.6195225 |
| RT | 110 | 0.309033232 | 0.2635491 | -0.20838987 | 0.139737117 | 0.3059665 | 0.4739554 | 0.852074 |
| RT | 170 | 0.334394855 | 0.3298429 | -0.308336903 | 0.124875847 | 0.32779607 | 0.5407049 | 1.0108954 |
| RT | 220 | 0.327982263 | 0.3564719 | -0.373084447 | 0.104742085 | 0.32334067 | 0.5508887 | 1.0472751 |
| RT | 280 | 0.287230419 | 0.3571481 | -0.426404489 | 0.060670765 | 0.28239155 | 0.5139503 | 1.0086828 |
| RT | 330 | 0.22572327 | 0.3392048 | -0.441099117 | 0.009156382 | 0.21407986 | 0.4367041 | 0.9177959 |
| RT | 390 | 0.118857957 | 0.3183099 | -0.507801713 | -0.093877369 | 0.11774099 | 0.3177094 | 0.7659507 |
| RT | 440 | 0.002256252 | 0.3349437 | -0.665285183 | -0.22448452 | 0.01186923 | 0.2206012 | 0.6829517 |
| RT | 500 | -0.170722529 | 0.4353262 | -1.034121115 | -0.450411243 | -0.16116336 | 0.1132513 | 0.6644755 |
| Walking AE | 0 | 0.168876176 | 0.1063261 | 0.018734399 | 0.087919555 | 0.15001639 | 0.230073 | 0.4185282 |
| Walking AE | 83 | 0.2624625 | 0.1369332 | 0.022719362 | 0.165867916 | 0.2515479 | 0.3467628 | 0.5666948 |
| Walking AE | 170 | 0.341438051 | 0.1842485 | -0.005281485 | 0.216696791 | 0.33539946 | 0.4572483 | 0.7232212 |
| Walking AE | 250 | 0.39678704 | 0.214909 | -0.01097866 | 0.254154296 | 0.39001249 | 0.5301406 | 0.8430487 |
| Walking AE | 330 | 0.435587804 | 0.2298494 | -0.001545856 | 0.285612257 | 0.42834991 | 0.5786426 | 0.9092412 |
| Walking AE | 420 | 0.459458364 | 0.2320523 | 0.014909507 | 0.309918456 | 0.45235326 | 0.6038976 | 0.9333919 |
| Walking AE | 500 | 0.463094152 | 0.2314145 | 0.020033711 | 0.315541599 | 0.45456805 | 0.6083086 | 0.9425991 |
| Walking AE | 580 | 0.450181715 | 0.2459338 | -0.018913839 | 0.291737472 | 0.44598663 | 0.6067136 | 0.9516012 |
| Walking AE | 670 | 0.415874925 | 0.3032059 | -0.164754693 | 0.216187381 | 0.41058507 | 0.613331 | 1.0262521 |
| Walking AE | 750 | 0.367797512 | 0.3982456 | -0.401396376 | 0.107439967 | 0.3642174 | 0.6322708 | 1.162998 |

Table S11. Predicted effects of exercise modality–dose combinations on working memory (MBNMA estimates).

| agent | dose | mean | sd | 2.5% | 25% | 50% | 75% | 97.5% |
| --- | --- | --- | --- | --- | --- | --- | --- | --- |
| Placebo | 0 | 0.1661087 | 0.1005722 | 0.025091646 | 0.089790584 | 0.1461973 | 0.2274229 | 0.4021916 |
| CPT | 0 | 0.1661087 | 0.1005722 | 0.025091646 | 0.089790584 | 0.1461973 | 0.2274229 | 0.4021916 |
| CPT | 110 | 0.1687897 | 0.11626 | -0.020092155 | 0.085785165 | 0.1587284 | 0.241235 | 0.4288932 |
| CPT | 220 | 0.1726069 | 0.1408253 | -0.081528451 | 0.07562266 | 0.1655906 | 0.2627243 | 0.4687518 |
| CPT | 330 | 0.1775602 | 0.1588535 | -0.117343259 | 0.069444062 | 0.1724338 | 0.2778119 | 0.5064208 |
| CPT | 440 | 0.1836497 | 0.166383 | -0.126621399 | 0.070899594 | 0.1797216 | 0.2890321 | 0.5254509 |
| CPT | 560 | 0.1915886 | 0.1635025 | -0.109540036 | 0.0779556 | 0.1888754 | 0.2952892 | 0.5239823 |
| CPT | 670 | 0.2000537 | 0.1558108 | -0.089105946 | 0.093573445 | 0.1995681 | 0.2997134 | 0.5248494 |
| CPT | 780 | 0.209655 | 0.15442 | -0.081692719 | 0.104727684 | 0.2031542 | 0.3151452 | 0.5308311 |
| CPT | 890 | 0.2203924 | 0.1752173 | -0.106184094 | 0.098146492 | 0.216729 | 0.337501 | 0.5751157 |
| CPT | 1000 | 0.232266 | 0.227797 | -0.197524191 | 0.067408558 | 0.2309529 | 0.3844638 | 0.6914726 |
| EJCT | 0 | 0.1661087 | 0.1005722 | 0.025091646 | 0.089790584 | 0.1461973 | 0.2274229 | 0.4021916 |
| EJCT | 83 | 0.2342173 | 0.1152514 | 0.043393851 | 0.149364304 | 0.2195673 | 0.3090755 | 0.4914836 |
| EJCT | 170 | 0.2882787 | 0.1416784 | 0.031133312 | 0.186411938 | 0.2811556 | 0.3798164 | 0.5852728 |
| EJCT | 250 | 0.3223363 | 0.1607366 | 0.028955015 | 0.208511386 | 0.3180579 | 0.4271037 | 0.6535004 |
| EJCT | 330 | 0.341396 | 0.1704107 | 0.027342072 | 0.220404005 | 0.3366861 | 0.4501326 | 0.6917696 |
| EJCT | 420 | 0.344911 | 0.1693369 | 0.034349058 | 0.225926821 | 0.3410604 | 0.4510948 | 0.6914618 |
| EJCT | 500 | 0.3321001 | 0.1594846 | 0.047120443 | 0.220519142 | 0.3284924 | 0.4317112 | 0.6666292 |
| EJCT | 580 | 0.3042914 | 0.1461681 | 0.044608942 | 0.199632947 | 0.2980366 | 0.3935728 | 0.6181079 |
| EJCT | 670 | 0.2550794 | 0.140451 | 0.009972539 | 0.154767762 | 0.2463506 | 0.3452552 | 0.5583393 |
| EJCT | 750 | 0.1954001 | 0.1606243 | -0.094699311 | 0.084832839 | 0.1871479 | 0.2997245 | 0.5269166 |
| MBE | 0 | 0.1661087 | 0.1005722 | 0.025091646 | 0.089790584 | 0.1461973 | 0.2274229 | 0.4021916 |
| MBE | 83 | 0.1610181 | 0.1110766 | -0.012707636 | 0.07923436 | 0.14471 | 0.2325492 | 0.4092259 |
| MBE | 170 | 0.1588119 | 0.1287499 | -0.063992518 | 0.067963339 | 0.1491599 | 0.2424779 | 0.4345813 |
| MBE | 250 | 0.1596101 | 0.1415849 | -0.09550134 | 0.059497576 | 0.1501196 | 0.2523937 | 0.4588025 |
| MBE | 330 | 0.1631168 | 0.1483203 | -0.104870053 | 0.059844298 | 0.1532202 | 0.2602069 | 0.4725898 |
| MBE | 420 | 0.1702994 | 0.1489649 | -0.103995413 | 0.067265511 | 0.1621972 | 0.2666151 | 0.4802303 |
| MBE | 500 | 0.1795618 | 0.146313 | -0.088268789 | 0.07881919 | 0.1715513 | 0.2741144 | 0.4869526 |
| MBE | 580 | 0.1915326 | 0.1464505 | -0.074456252 | 0.091502439 | 0.1824079 | 0.285056 | 0.500007 |
| MBE | 670 | 0.2082372 | 0.1602873 | -0.090079885 | 0.098942951 | 0.2015393 | 0.3085961 | 0.5386388 |
| MBE | 750 | 0.2259636 | 0.1924943 | -0.131436994 | 0.096265911 | 0.2239579 | 0.347164 | 0.6175388 |
| RT | 0 | 0.1661087 | 0.1005722 | 0.025091646 | 0.089790584 | 0.1461973 | 0.2274229 | 0.4021916 |
| RT | 56 | 0.2062874 | 0.1414719 | -0.060771633 | 0.110695929 | 0.1970693 | 0.2964643 | 0.5027018 |
| RT | 110 | 0.2389266 | 0.1981585 | -0.170156441 | 0.105725844 | 0.2357454 | 0.3721485 | 0.6370114 |
| RT | 170 | 0.2681631 | 0.2440983 | -0.250876626 | 0.109494522 | 0.2694575 | 0.4316368 | 0.7537442 |
| RT | 220 | 0.2868745 | 0.2636174 | -0.282526922 | 0.11627628 | 0.2877032 | 0.4623837 | 0.8028394 |
| RT | 280 | 0.3025454 | 0.2637076 | -0.261901026 | 0.12998017 | 0.3040759 | 0.4752784 | 0.8181096 |
| RT | 330 | 0.3099522 | 0.2455487 | -0.206983342 | 0.147846166 | 0.3126589 | 0.471089 | 0.7883451 |
| RT | 390 | 0.3120576 | 0.2072663 | -0.108238909 | 0.174360615 | 0.3117737 | 0.4487214 | 0.7128386 |
| RT | 440 | 0.3081598 | 0.1744403 | -0.023841674 | 0.187486012 | 0.3070653 | 0.421656 | 0.6639013 |
| RT | 500 | 0.2966996 | 0.1758578 | -0.044071539 | 0.179961834 | 0.2984014 | 0.4129147 | 0.6451851 |
| VRT | 0 | 0.1661087 | 0.1005722 | 0.025091646 | 0.089790584 | 0.1461973 | 0.2274229 | 0.4021916 |
| VRT | 110 | 0.1590077 | 0.1492677 | -0.10952072 | 0.0531 | 0.152814 | 0.2561606 | 0.4777093 |
| VRT | 220 | 0.1602355 | 0.2134392 | -0.235208195 | 0.01211677 | 0.156878 | 0.3061496 | 0.594073 |
| VRT | 330 | 0.169792 | 0.2570928 | -0.307139272 | -0.007740474 | 0.1660231 | 0.3476336 | 0.6722259 |
| VRT | 440 | 0.1876774 | 0.2754382 | -0.336253919 | -0.000561003 | 0.1827453 | 0.3774103 | 0.7299732 |
| VRT | 560 | 0.2166877 | 0.2679122 | -0.309055813 | 0.038391076 | 0.2138483 | 0.39987 | 0.7385575 |
| VRT | 670 | 0.2519879 | 0.2421811 | -0.223018269 | 0.096840594 | 0.2511571 | 0.4109987 | 0.728398 |
| VRT | 780 | 0.2956169 | 0.2159599 | -0.123686513 | 0.151275886 | 0.2945955 | 0.433122 | 0.7262485 |
| VRT | 890 | 0.3475746 | 0.2268548 | -0.080938607 | 0.195944661 | 0.343886 | 0.491882 | 0.8166513 |
| VRT | 1000 | 0.4078612 | 0.307666 | -0.19196269 | 0.2108845 | 0.4047924 | 0.6064427 | 1.0434492 |
| Walking AE | 0 | 0.1661087 | 0.1005722 | 0.025091646 | 0.089790584 | 0.1461973 | 0.2274229 | 0.4021916 |
| Walking AE | 56 | 0.2471063 | 0.141674 | -0.000495434 | 0.145091106 | 0.2369872 | 0.338845 | 0.5419087 |
| Walking AE | 110 | 0.306281 | 0.1985537 | -0.056192861 | 0.166458925 | 0.3004321 | 0.4333733 | 0.7094106 |
| Walking AE | 170 | 0.3502324 | 0.2449641 | -0.102679728 | 0.177518884 | 0.3431018 | 0.5105534 | 0.837271 |
| Walking AE | 220 | 0.3693307 | 0.2649506 | -0.121763582 | 0.181906917 | 0.3615437 | 0.5412766 | 0.9020892 |
| Walking AE | 280 | 0.3712152 | 0.265301 | -0.120662517 | 0.1831549 | 0.362687 | 0.5474492 | 0.9063017 |
| Walking AE | 330 | 0.3552578 | 0.2464344 | -0.097944412 | 0.179753073 | 0.3483077 | 0.5164841 | 0.866911 |
| Walking AE | 390 | 0.3150754 | 0.2040628 | -0.054457832 | 0.172084099 | 0.3065781 | 0.44268 | 0.7343716 |
| Walking AE | 440 | 0.2640622 | 0.1610948 | -0.020127226 | 0.151407515 | 0.253152 | 0.3674565 | 0.5957273 |
| Walking AE | 500 | 0.1818129 | 0.1400383 | -0.069688816 | 0.086490293 | 0.1713542 | 0.2716584 | 0.477499 |

Table S12. Predicted effects of exercise modality–dose combinations on inhibitory control (MBNMA estimates).

| agent | dose | mean | sd | 2.5% | 25% | 50% | 75% | 97.5% |
| --- | --- | --- | --- | --- | --- | --- | --- | --- |
| Placebo | 0 | 0.165222106 | 0.1023071 | 0.022338831 | 0.08719844 | 0.145677983 | 0.2254781 | 0.4027848 |
| CPT | 0 | 0.165222106 | 0.1023071 | 0.022338831 | 0.08719844 | 0.145677983 | 0.2254781 | 0.4027848 |
| CPT | 110 | 0.382469009 | 0.189807 | 0.017677482 | 0.25832644 | 0.378049325 | 0.5018957 | 0.7734203 |
| CPT | 220 | 0.547789407 | 0.2927473 | -0.028534085 | 0.36529433 | 0.550354102 | 0.7317371 | 1.1379999 |
| CPT | 330 | 0.661183301 | 0.3595757 | -0.059666102 | 0.44202594 | 0.665402332 | 0.8859142 | 1.3937379 |
| CPT | 440 | 0.722650691 | 0.3857055 | -0.063836982 | 0.48805149 | 0.72704227 | 0.9637255 | 1.5100623 |
| CPT | 560 | 0.730484061 | 0.3686679 | -0.0026632 | 0.50130757 | 0.736251661 | 0.9629726 | 1.4851683 |
| CPT | 670 | 0.683377849 | 0.3165911 | 0.055932906 | 0.48611015 | 0.680794616 | 0.8821442 | 1.3215817 |
| CPT | 780 | 0.584345134 | 0.2480303 | 0.107308345 | 0.42579774 | 0.581081455 | 0.7469488 | 1.0724253 |
| CPT | 890 | 0.433385914 | 0.226875 | -0.010341275 | 0.28623017 | 0.432520969 | 0.5811181 | 0.8863679 |
| CPT | 1000 | 0.230500189 | 0.3375412 | -0.433563822 | 0.01888183 | 0.23265799 | 0.4372962 | 0.8959631 |
| EJCT | 0 | 0.165222106 | 0.1023071 | 0.022338831 | 0.08719844 | 0.145677983 | 0.2254781 | 0.4027848 |
| EJCT | 83 | 0.250978285 | 0.2067168 | -0.126943596 | 0.11527961 | 0.237537747 | 0.3833053 | 0.692389 |
| EJCT | 170 | 0.314929142 | 0.3262629 | -0.304337367 | 0.10081576 | 0.299329132 | 0.5261535 | 0.9794625 |
| EJCT | 250 | 0.350304257 | 0.3944594 | -0.394343723 | 0.08958676 | 0.333753146 | 0.5989678 | 1.1585367 |
| EJCT | 330 | 0.36323121 | 0.4201726 | -0.414824546 | 0.08794226 | 0.34619223 | 0.6293643 | 1.2212745 |
| EJCT | 420 | 0.350941463 | 0.4026946 | -0.425329579 | 0.07698178 | 0.337600281 | 0.6096035 | 1.1686013 |
| EJCT | 500 | 0.316166071 | 0.3582965 | -0.381033024 | 0.07520687 | 0.301165718 | 0.5402038 | 1.0647744 |
| EJCT | 580 | 0.258942516 | 0.3198812 | -0.3655764 | 0.04676052 | 0.249010362 | 0.4650759 | 0.9036644 |
| EJCT | 670 | 0.167733447 | 0.3663996 | -0.546795609 | -0.06368646 | 0.174364591 | 0.396356 | 0.8973226 |
| EJCT | 750 | 0.062807546 | 0.5289238 | -0.991588264 | -0.27639746 | 0.075899171 | 0.3954519 | 1.0840123 |
| MBE | 0 | 0.165222106 | 0.1023071 | 0.022338831 | 0.08719844 | 0.145677983 | 0.2254781 | 0.4027848 |
| MBE | 83 | 0.19512039 | 0.1695528 | -0.13432013 | 0.08369838 | 0.188824383 | 0.3025128 | 0.5462453 |
| MBE | 170 | 0.222090429 | 0.2604036 | -0.312885468 | 0.0588545 | 0.219169893 | 0.3902949 | 0.7416784 |
| MBE | 250 | 0.242943775 | 0.3165556 | -0.4056108 | 0.04826148 | 0.241224986 | 0.4479538 | 0.8704219 |
| MBE | 330 | 0.260015863 | 0.3418915 | -0.436662828 | 0.05154587 | 0.256164844 | 0.482363 | 0.9349487 |
| MBE | 420 | 0.274702175 | 0.3344178 | -0.406305193 | 0.06935671 | 0.268910398 | 0.486034 | 0.9205303 |
| MBE | 500 | 0.283739087 | 0.3005008 | -0.321680215 | 0.10338728 | 0.280489522 | 0.4710542 | 0.8693757 |
| MBE | 580 | 0.28899474 | 0.2544051 | -0.211394947 | 0.13210804 | 0.283458699 | 0.4472787 | 0.7871686 |
| MBE | 670 | 0.290387563 | 0.2354381 | -0.17690672 | 0.14306785 | 0.287907754 | 0.4361252 | 0.750654 |
| MBE | 750 | 0.287608041 | 0.3077679 | -0.303488732 | 0.09250879 | 0.288249512 | 0.4776273 | 0.8879111 |
| RT | 0 | 0.165222106 | 0.1023071 | 0.022338831 | 0.08719844 | 0.145677983 | 0.2254781 | 0.4027848 |
| RT | 56 | 0.25921897 | 0.176513 | -0.066091994 | 0.14505173 | 0.250724379 | 0.368735 | 0.6284192 |
| RT | 110 | 0.318256268 | 0.2660541 | -0.191302282 | 0.14881991 | 0.31180224 | 0.4784188 | 0.8601561 |
| RT | 170 | 0.347462467 | 0.3335348 | -0.308031511 | 0.14021434 | 0.340521817 | 0.5469576 | 1.0161905 |
| RT | 220 | 0.342539358 | 0.3608791 | -0.374831109 | 0.11823832 | 0.333599688 | 0.5598929 | 1.071871 |
| RT | 280 | 0.301517699 | 0.3624144 | -0.421963669 | 0.0763839 | 0.295259869 | 0.5274724 | 1.029133 |
| RT | 330 | 0.238071376 | 0.3454702 | -0.43352753 | 0.01651972 | 0.231719274 | 0.4578624 | 0.9269807 |
| RT | 390 | 0.12682186 | 0.3264468 | -0.533567026 | -0.07757081 | 0.121135542 | 0.3328789 | 0.7917626 |
| RT | 440 | 0.004852322 | 0.3449096 | -0.677512068 | -0.21365098 | 0.001473813 | 0.2267253 | 0.6961083 |
| RT | 500 | -0.176625053 | 0.4469046 | -1.051211718 | -0.45082869 | -0.181292907 | 0.1034917 | 0.7271968 |
| Walking AE | 0 | 0.165222106 | 0.1023071 | 0.022338831 | 0.08719844 | 0.145677983 | 0.2254781 | 0.4027848 |
| Walking AE | 83 | 0.255169406 | 0.1349321 | 0.012570601 | 0.16274614 | 0.242516569 | 0.3370697 | 0.542826 |
| Walking AE | 170 | 0.331512383 | 0.1822457 | -0.019953495 | 0.21158677 | 0.32527627 | 0.4482849 | 0.7006787 |
| Walking AE | 250 | 0.385508168 | 0.2120681 | -0.025735191 | 0.24336738 | 0.378245194 | 0.5212345 | 0.8168838 |
| Walking AE | 330 | 0.423978538 | 0.2259059 | -0.005245719 | 0.27505239 | 0.415546941 | 0.5706784 | 0.8761099 |
| Walking AE | 420 | 0.448699981 | 0.2270515 | 0.009627922 | 0.29573487 | 0.438803259 | 0.5990448 | 0.9038994 |
| Walking AE | 500 | 0.454178842 | 0.2264047 | 0.01859659 | 0.29746262 | 0.448204378 | 0.6043809 | 0.9085685 |
| Walking AE | 580 | 0.444132287 | 0.2427889 | -0.014570447 | 0.27839048 | 0.44381555 | 0.6057973 | 0.927589 |
| Walking AE | 670 | 0.41427219 | 0.3043184 | -0.186737171 | 0.21080634 | 0.417771955 | 0.6183114 | 1.0197365 |

Table S13. Certainty of Evidence (GRADE) for Executive Function Outcomes

| **Outcome** | **No. of studies** | **Risk of Bias** | **Inconsistency** | **Indirectness** | **Imprecision** | **Publication Bias** | **Certainty of Evidence (GRADE)** |
| --- | --- | --- | --- | --- | --- | --- | --- |
| Executive Function (overall) | 38 | Downgraded by one level due to moderate PEDro scores and lack of blinding | No serious concern | No serious concern | Downgraded by one level due to small sample sizes | Possible | Moderate |
| Working Memory | 25 | Downgraded by one level due to moderate methodological quality | No serious concern | No serious concern | Downgraded by one level | Possible | Moderate |
| Cognitive Flexibility | 30 | Downgraded by one level | Downgraded by one level because of variability across studies | No serious concern | Downgraded by one level | Likely | Low to Moderate |
| Inhibitory Control | 17 | Downgraded by one level | Downgraded by one level due to inconsistent effect directions | No serious concern | Downgraded by one level because evidence base is small | Likely | Low |

Included studies: ^[1-38]^

[1]	KASAI J Y T, BUSSE A L, MAGALDI R M, et al. Effects of Tai Chi Chuan on cognition of elderly women with mild cognitive impairment [J]. Einstein (São Paulo), 2010, 8(1): 40-5.<https://doi.org/10.1590/s1679-45082010ao1470>

[2]	LAM L C W, CHAU R C M, WONG B M L, et al. Interim follow-up of a randomized controlled trial comparing Chinese style mind body (Tai Chi) and stretching exercises on cognitive function in subjects at risk of progressive cognitive decline [J]. International Journal of Geriatric Psychiatry, 2011, 26(7): 733-40.<https://doi.org/10.1002/gps.2602>

[3]	SUZUKI T, SHIMADA H, MAKIZAKO H, et al. Effects of multicomponent exercise on cognitive function in older adults with amnestic mild cognitive impairment: a randomized controlled trial [J]. Bmc Neurology, 2012, 12.<https://doi.org/10.1186/1471-2377-12-128>

[4]	BARNES D E, SANTOS-MODESITT W, POELKE G, et al. The Mental Activity and eXercise (MAX) Trial <i>A Randomized Controlled Trial to Enhance Cognitive Function in Older Adults</i> [J]. Jama Internal Medicine, 2013, 173(9): 797-804.<https://doi.org/10.1001/jamainternmed.2013.189>

[5]	DAVIS J C, BRYAN S, MARRA C A, et al. An Economic Evaluation of Resistance Training and Aerobic Training versus Balance and Toning Exercises in Older Adults with Mild Cognitive Impairment [J]. Plos One, 2013, 8(5).<https://doi.org/10.1371/journal.pone.0063031>

[6]	DANNHAUSER T M, CLEVERLEY M, WHITFIELD T J, et al. A complex multimodal activity intervention to reduce the risk of dementia in mild cognitive impairment-ThinkingFit: pilot and feasibility study for a randomized controlled trial [J]. Bmc Psychiatry, 2014, 14.<https://doi.org/10.1186/1471-244x-14-129>

[7]	LAM L C-W, CHAN W C, LEUNG T, et al. Would Older Adults with Mild Cognitive Impairment Adhere to and Benefit from a Structured Lifestyle Activity Intervention to Enhance Cognition?: A Cluster Randomized Controlled Trial [J]. Plos One, 2015, 10(3).<https://doi.org/10.1371/journal.pone.0118173>

[8]	LÜ J J, SUN M Y, LIANG L C, et al. Effects of momentum-based dumbbell training on cognitive function in older adults with mild cognitive impairment: a pilot randomized controlled trial [J]. Clinical Interventions in Aging, 2016, 11: 9-16.<https://doi.org/10.2147/cia.S96042>

[9]	DOI T, VERGHESE J, MAKIZAKO H, et al. Effects of Cognitive Leisure Activity on Cognition in Mild Cognitive Impairment: Results of a Randomized Controlled Trial [J]. Journal of the American Medical Directors Association, 2017, 18(8): 686-91.<https://doi.org/10.1016/j.jamda.2017.02.013>

[10]	EYRE H A, SIDDARTH P, ACEVEDO B, et al. A randomized controlled trial of Kundalini yoga in mild cognitive impairment [J]. International Psychogeriatrics, 2017, 29(4): 557-67.<https://doi.org/10.1017/s1041610216002155>

[11]	HWANG J, LEE S. The effect of virtual reality program on the cognitive function and balance of the people with mild cognitive impairment [J]. Journal of Physical Therapy Science, 2017, 29(8): 1283-6.

[12]	COMBOURIEU DONNEZAN L, PERROT A, BELLEVILLE S, et al. Effects of simultaneous aerobic and cognitive training on executive functions, cardiovascular fitness and functional abilities in older adults with mild cognitive impairment [J]. Mental Health and Physical Activity, 2018, 15: 78-87.<https://doi.org/https://doi.org/10.1016/j.mhpa.2018.06.001>

[13]	DAMIRCHI A, HOSSEINI F, BABAEI P. Mental Training Enhances Cognitive Function and BDNF More Than Either Physical or Combined Training in Elderly Women With MCI: A Small-Scale Study [J]. American Journal of Alzheimers Disease and Other Dementias, 2018, 33(1): 20-9.<https://doi.org/10.1177/1533317517727068>

[14]	HONG S-G, KIM J-H, JUN T-W. Effects of 12-Week Resistance Exercise on Electroencephalogram Patterns and Cognitive Function in the Elderly With Mild Cognitive Impairment: A Randomized Controlled Trial [J]. Clinical Journal of Sport Medicine, 2018, 28(6): 500-8.<https://doi.org/10.1097/jsm.0000000000000476>

[15]	SUNGKARAT S, BORIPUNTAKUL S, KUMFU S, et al. Tai Chi Improves Cognition and Plasma BDNF in Older Adults With Mild Cognitive Impairment: A Randomized Controlled Trial [J]. Neurorehabilitation and Neural Repair, 2018, 32(2): 142-9.<https://doi.org/10.1177/1545968317753682>

[16]	YOON D H, LEE J Y, SONG W. EFFECTS OF RESISTANCE EXERCISE TRAINING ON COGNITIVE FUNCTION AND PHYSICAL PERFORMANCE IN COGNITIVE FRAILTY: A RANDOMIZED CONTROLLED TRIAL [J]. Journal of Nutrition Health & Aging, 2018, 22(8): 944-51.<https://doi.org/10.1007/s12603-018-1090-9>

[17]	LAW L L F, MOK V C T, YAU M M K. Effects of functional tasks exercise on cognitive functions of older adults with mild cognitive impairment: a randomized controlled pilot trial [J]. Alzheimers Research & Therapy, 2019, 11(1).<https://doi.org/10.1186/s13195-019-0548-2>

[18]	LIAO Y-Y, CHEN I H, LIN Y-J, et al. Effects of Virtual Reality-Based Physical and Cognitive Training on Executive Function and Dual-Task Gait Performance in Older Adults With Mild Cognitive Impairment: A Randomized Control Trial [J]. Frontiers in Aging Neuroscience, 2019, 11.<https://doi.org/10.3389/fnagi.2019.00162>

[19]	PARK E, YUN B-J, MIN Y-S, et al. Effects of a Mixed Reality-based Cognitive Training System Compared to a Conventional Computer-assisted Cognitive Training System on Mild Cognitive Impairment: A Pilot Study [J]. Cognitive and Behavioral Neurology, 2019, 32(3): 172-8.<https://doi.org/10.1097/wnn.0000000000000197>

[20]	XIA R, QIU P T, LIN H Y, et al. The Effect of Traditional Chinese Mind-Body Exercise (Baduanjin) and Brisk Walking on the Dorsal Attention Network in Older Adults With Mild Cognitive Impairment [J]. Frontiers in Psychology, 2019, 10.<https://doi.org/10.3389/fpsyg.2019.02075>

[21]	MANENTI R, GOBBI E, BAGLIO F, et al. Effectiveness of an Innovative Cognitive Treatment and Telerehabilitation on Subjects With Mild Cognitive Impairment: A Multicenter, Randomized, Active-Controlled Study [J]. Frontiers in Aging Neuroscience, 2020, 12.<https://doi.org/10.3389/fnagi.2020.585988>

[22]	PARK J-S, JUNG Y-J, LEE G. Virtual Reality-Based Cognitive-Motor Rehabilitation in Older Adults with Mild Cognitive Impairment: A Randomized Controlled Study on Motivation and Cognitive Function [J]. Healthcare, 2020, 8(3).<https://doi.org/10.3390/healthcare8030335>

[23]	THAPA N, PARK H J, YANG J-G, et al. The Effect of a Virtual Reality-Based Intervention Program on Cognition in Older Adults with Mild Cognitive Impairment: A Randomized Control Trial [J]. Journal of Clinical Medicine, 2020, 9(5).<https://doi.org/10.3390/jcm9051283>

[24]	LI P W C, YU D S F, SIU P M, et al. Peer-supported exercise intervention for persons with mild cognitive impairment: a waitlist randomised controlled trial (the BRAin Vitality Enhancement trial) [J]. Age and Ageing, 2022, 51(10).<https://doi.org/10.1093/ageing/afac213>

[25]	XIA R, WAN M Y, LIN H Y, et al. Effects of mind-body exercise Baduanjin on cognition in community-dwelling older people with mild cognitive impairment: A randomized controlled trial [J]. Neuropsychological Rehabilitation, 2023, 33(8): 1368-83.<https://doi.org/10.1080/09602011.2022.2099909>

[26]	FAIRCHILD J K, MYERS J, LOURAS P, et al. Multimodal Exercise and Cognitive Training Program Improves Cognitive Function in Amnestic Mild Cognitive Impairment [J]. American Journal of Geriatric Psychiatry, 2024, 32(4): 463-74.<https://doi.org/10.1016/j.jagp.2023.12.002>

[27]	FAN M X, LI Q F, YANG T T, et al. Effect of Multimodal Intervention in Individuals with Mild Cognitive Impairment: A Randomized Clinical Trial in Shanghai [J]. Journal of Alzheimers Disease, 2024, 101(1): 235-48.<https://doi.org/10.3233/jad-231370>

[28]	KROOTNARK K, CHAIKEEREE N, SAENGSIRISUWAN V, et al. Effects of low-intensity home-based exercise on cognition in older persons with mild cognitive impairment: a direct comparison of aerobic versus resistance exercises using a randomized controlled trial design [J]. Frontiers in Medicine, 2024, 11.<https://doi.org/10.3389/fmed.2024.1392429>

[29]	SAEED A, NIAZI I K, ALZAHRANI A, et al. A difficulty based comparison of novel exergame balance training for cognitive functions in adults with mild cognitive impairment: A randomized trial [J]. Mental Health and Physical Activity, 2024, 27.<https://doi.org/10.1016/j.mhpa.2024.100637>

[30]	WANG H, PEI Z, LIU Y. Effects of square dance exercise on cognitive function in elderly individuals with mild cognitive impairment: the mediating role of balance ability and executive function [J]. BMC Geriatrics, 2024, 24.<https://doi.org/10.1186/s12877-024-04714-x>

[31]	BAKER L D, PA J A, KATULA J A, et al. Effects of exercise on cognition and Alzheimer's biomarkers in a randomized controlled trial of adults with mild cognitive impairment: The EXERT study [J]. Alzheimers & Dementia, 2025, 21(4).<https://doi.org/10.1002/alz.14586>

[32]	IP W K, SOAR J, JAMES C, et al. Virtual reality game-based training for preventing falls among community-dwelling older adults with mild cognitive impairment: a pilot randomized control trial study [J]. Virtual Reality, 2025, 29(1).<https://doi.org/10.1007/s10055-024-01084-y>

[33]	MEEKUM S, YUENYONGCHAIWAT K, LUANGPON N, et al. Enhancing Global Cognition and Executive Functions Through Water-Based Exercise in Mild Cognitive Impairment: A Randomized Controlled Trial [J]. Life-Basel, 2025, 15(3).<https://doi.org/10.3390/life15030420>

[34]	MUÑOZ-PERETE J M, CARCELÉN-FRAILE M D, CASTELLOTE-CABALLERO Y, et al. Cognitive Stimulation and Strength Training in Older Adults with Mild Cognitive Impairment: A Randomized Controlled Trial [J]. Diagnostics, 2025, 15(12).<https://doi.org/10.3390/diagnostics15121477>

[35]	PENUKONDA S, SRINIVASAN S, TARUMI T, et al. One-year exercise improves cognition and fitness and decreases vascular stiffness and reactivity to CO2 in amnestic mild cognitive impairment [J]. Journal of Alzheimers Disease, 2025, 105(1): 245-57.<https://doi.org/10.1177/13872877251325575>

[36]	RESENDE-SILVA S, DE RESENDE-NETO A G, VASCONCELOS A B S, et al. Functional training improves cognitive function, functional fitness, and BDNF levels in older women with mild cognitive impairment: a randomized controlled trial [J]. Frontiers in Physiology, 2025, 16.<https://doi.org/10.3389/fphys.2025.1638590>

[37]	SÁNCHEZ-ALCALA M, AIBAR-ALMAZÁN A, CARCELÉN-FRAILE M D, et al. Effects of Dance-Based Aerobic Training on Frailty and Cognitive Function in Older Adults with Mild Cognitive Impairment: A Randomized Controlled Trial [J]. Diagnostics, 2025, 15(3).<https://doi.org/10.3390/diagnostics15030351>

[38]	YU H, MA B X, FENG Y C, et al. Effects of multi-domain cognitive-motor training in older adults with amnestic mild cognitive impairment: A randomized controlled trial [J]. Geriatric Nursing, 2025, 61: 64-72.<https://doi.org/10.1016/j.gerinurse.2024.10.072>
