# Supplementary material for: Optimizing exercise prescriptions for older adults with mild cognitive impairment: a dose–response network meta-analysis of modality–dose combinations on executive function
Source: PeerJ. 2026 Oct 20;14:e21805. doi: 10.7717/peerj.21805 (PMC13693602; doi:10.7717/peerj.21805)
Supplement: Supplementary file 9 [file peerj-14-21805-s009.docx]

**1. Audience**
This systematic review and Bayesian network meta-analysis is intended for clinicians, rehabilitation practitioners, exercise specialists, and researchers involved in the management and study of age-related cognitive decline. It is particularly relevant to those designing and implementing exercise-based programmes for older adults with mild cognitive impairment, as well as to exercise scientists and cognitive neuroscientists interested in the dose–response relationship between physical activity and executive function. The findings may also inform public health professionals and policymakers involved in community-based cognitive health promotion. In addition, the identified optimal dose ranges and modality–dose combinations may help guide the design of future randomized controlled trials and support greater standardization of exercise interventions.

**2. Resolution of disagreements and referee**
During data processing, Ming-liang Song and Qiang Xiong independently screened all candidate studies according to the predefined inclusion and exclusion criteria and extracted the required information. Any disagreements were first resolved through discussion. If consensus could not be reached, Xiao-jin Qin acted as the independent referee and made the final decision.
